# Evaluation of a Collagen-Chitosan Hydrogel for Potential Use as a Pro-Angiogenic Site for Islet Transplantation

**DOI:** 10.1371/journal.pone.0077538

**Published:** 2013-10-18

**Authors:** Joanne E. McBane, Branka Vulesevic, Donna T. Padavan, Kimberly A. McEwan, Gregory S. Korbutt, Erik J. Suuronen

**Affiliations:** 1 University of Ottawa Heart Institute, Ottawa, Canada; 2 Department of Cellular & Molecular Medicine, University of Ottawa, Ottawa, Canada; 3 Faculty of Engineering, University of Ottawa, Ottawa, Canada; 4 Alberta Diabetes Institute, University of Alberta, Edmonton, Canada; University of Bremen, Germany

## Abstract

Islet transplantation to treat type 1 diabetes (T1D) has shown varied long-term success, due in part to insufficient blood supply to maintain the islets. In the current study, collagen and collagen:chitosan (10:1) hydrogels, +/- circulating angiogenic cells (CACs), were compared for their ability to produce a pro-angiogenic environment in a streptozotocin-induced mouse model of T1D. Initial characterization showed that collagen-chitosan gels were mechanically stronger than the collagen gels (0.7kPa vs. 0.4kPa elastic modulus, respectively), had more cross-links (9.2 vs. 7.4/µm^2^), and were degraded more slowly by collagenase. After gelation with CACs, live/dead staining showed greater CAC viability in the collagen-chitosan gels after 18h compared to collagen (79% vs. 69%). *In vivo*, collagen-chitosan gels, subcutaneously implanted for up to 6 weeks in a T1D mouse, showed increased levels of pro-angiogenic cytokines over time. By 6 weeks, anti-islet cytokine levels were decreased in all matrix formulations ± CACs. The 6-week implants demonstrated increased expression of VCAM-1 in collagen-chitosan implants. Despite this, infiltrating vWF^+^ and CXCR4^+^ angiogenic cell numbers were not different between the implant types, which may be due to a delayed and reduced cytokine response in a T1D versus non-diabetic setting. The mechanical, degradation and cytokine data all suggest that the collagen-chitosan gel may be a suitable candidate for use as a pro-angiogenic ectopic islet transplant site.

## Introduction

Islet transplantation has become an attractive therapy for type I diabetes (T1D). The Edmonton protocol has greatly increased the survival and initial function of transplanted islets in humans with T1D [1]. However, long-term islet survival remains sub-optimal and these patients only yielded an ~10% rate of insulin independence after 5 years [2]. Although portal vein injection of islets into the liver is the most common procedure for islet transplantation (and used in the Edmonton protocol), the need for a safer transplant site has been identified as an important issue to address [3-6]. 

An ideal transplant site should provide liberal access to oxygen and nutrients, as well as venous drainage for the control of blood glucose levels through insulin secretion. Therefore, a strategy for promoting angiogenesis at the transplant site may be necessary for islet grafting and function. Different approaches to vascularize transplant sites have been reported in diabetic animals. For example, a silicone tube/Matrigel groin chamber model [7], a poly(lactide-co-glycolide and poly-L-lactic acid hybrid scaffold [8], electrospun polymer mats [9], a subcutaneous hydrogel-type fibrin [10], a polymer cell pouch device [11], and a denuded intestinal segment [12] have all demonstrated vascularity and an ability to support islets. 

During angiogenesis, acute pro-inflammatory signaling is followed by a transition to an anti-inflammatory or wound-healing process. However, the inflammatory response may adversely affect islet grafting and function/survival *in vivo* (reviewed in 13). (Table S1in File S1) categorizes important cytokines that have been shown to have roles in both angiogenesis and islet graft survival. Ultimately, a balance may be needed, since the pro-angiogenic signaling necessary for vascularization include pro-inflammatory cytokines that can be detrimental to islet graft implantation. This consideration has received little attention in the development of ectopic islet transplant sites, but it has the potential to help optimize islet engraftment, survival and function. 

We have previously demonstrated that adding chitosan to collagen hydrogels can promote angiogenesis *in vitro* and *in vivo* in a non-diabetic model [14]. Therefore, the collagen-chitosan matrix combined with pro-angiogenic cells, such as circulating angiogenic cells (CACs), may provide an ideal environment for promoting a pro-angiogenic islet transplant site; however, our materials have not been tested in diabetic models, nor has the cytokine signaling they elicit been evaluated. 

In the current study, we evaluated collagen and collagen-chitosan hydrogels as potential pro-angiogenic sites for islet transplantation. The objectives were to: 1) determine if the addition of chitosan and/or CACs could enhance the suitability of the collagen matrix to serve as a pro-angiogenic ectopic islet transplant site in a mouse model of T1D (streptozotocin (STZ)-induced); and 2) characterize the cytokine milieu as a means of predicting the ideal time between hydrogel implantation and the introduction of islets. 

## Materials and Methods

### Ethics Statement

The protocol for blood procurement and CAC isolation was approved by the Human Research Ethics Board of the University of Ottawa Heart Institute and informed written consent was obtained from all volunteers. All animal studies were approved by the University of Ottawa Animal Care Committee, in compliance with the National Institute of Health’s Guide for the Care and Use of Laboratory Animals. 

### CAC Isolation

Approximately 100ml of blood was procured from healthy human volunteers and peripheral blood mononuclear cells (PBMCs) were isolated using Histopaque and density centrifugation. PBMCs were plated for 4d on fibronectin-coated plates to generate the heterogeneous population of CACs, as previously described [15]. 

### Preparation of Collagen-Chitosan Hydrogels

A 1% rat tail collagen type I solution (BD Biosciences) and a 1.5% chitosan (w/v) HCl solution (0.2M), both buffered with a 0.5M morpholinoethanesulfonic acid solution (MES) and NaOH (1N) to a pH of ~7.2, were mixed together at a 10:1 ratio (w/w). Collagen buffer (stock solution of 10× DMEM with 0.2M HEPES, 35% FBS, and gentamycin, pH 7.2)) was then added to the mixture, and represented 8% of the total gel volume. Aqueous solutions of 1-ethyl-3-(3-dimethyl aminopropyl) carbodiimide (EDC) and N-hydroxysuccinimide (NHS) (both at 10% (w/v) in MES; EDC:NHS to collagen-NH_2_ = 6 molar equivalents) were mixed with the collagen-chitosan solution, on ice. The solution was allowed to cross-link for 5min prior to pH adjustment (7.2-7.4), using MES and 1N NaOH. Glycine was then added, and mixed with or without CACs (1×10^7^ CACs per gel in a 6-well plate; final collagen concentration was 0.52mg/ml). After gelation for 30min at 37°C, complete endothelial basal medium (EBM, Clonetics) was added and the gels were returned to the incubator for 18-24h. To make collagen hydrogels, chitosan was omitted from the procedure. An 8mm biopsy punch was used to cut disk-shaped gels, which were implanted *in vivo* or subjected to *in vitro* testing.

### Degradation Study

The degradation rate of hydrogels was tested *in vitro* using collagenase and amylase. Collagenase I (Gibco) was tested at 0.1, 1, 10, 100 or 400 units/ml in phosphate buffered saline (PBS, pH 7.2) for up to 24h. Longer term degradation of the hydrogels (6 weeks) was tested with 0.1 units of collagenase. For α-Amylase (which degrades chitosan [16]), hydrogels were incubated in 750µl of stock α-amylase solution (1100 units/ml) for up to 6 weeks and compared to disks incubated in PBS alone. For all degradation studies lasting past 2 days, the enzyme solution was replaced three times per week. At various time points, the samples were weighed and the % of original mass remaining was calculated. The original mass was the mass of the biopsy-punched hydrogel after immersion in sterile PBS for 24h at 37°C.

### Critical Point Drying (CPD) and Scanning Electron Microscopy (SEM)

Hydrogels were fixed in 3% glutaraldehyde and prepared for SEM, as described in the Supplemental Methods (in File S1). Fiber length, fiber diameter and number of fiber intersections (cross-links) were quantified by evaluating representative images from 4 separate samples using Image-J software. 

### Mechanical Testing

Hydrogels were swelled for 48h in PBS at 37°C. Unconfined compression tests were performed using a servo-hydraulic material testing system (MTS Bionix 858) with a 5kg load cell at a cross speed of 50%/min and strained to a maximum of 65% strain. The stress-strain data was fitted for each sample (3mm thick) to a five-parameter double exponential growth model using: 

$$\sigma =y_{0}+a\cdot exp(b\cdot \varepsilon )+\text{c}\cdot exp(d\cdot \varepsilon )$$ (1)

where σ is stress, ε is strain, and y_0_, a, b,c and d are curve fitting parameters. The elastic modulus, as a function of strain was calculated by differentiating Equation (1) as follows:

$$\sigma^{\prime }=a\cdot b\cdot exp(b\cdot \varepsilon )+\text{c}\cdot d\cdot exp(d\cdot \varepsilon )$$ (2)

where *σ* ^′^ is the tangent modulus, ε is strain, and a, b, c and d are curve fitting parameters. The elastic modulus was calculated in the linear region of the stress-strain curve. Additional details are provided in the Supplemental Methods (in File S1).

### LIVE/DEAD Cell Viability Assay

The viability of CACs embedded in hydrogels was tested after 24h *in vitro* using the LIVE/DEAD^®^ kit (Invitrogen) as per manufacturer’s protocol. Briefly, after 24h, EBM was replaced with ethidium homodimer (1:500; red = dead) and calcein AM (1:2000; green = alive) in PBS for 30min in the dark. The samples were then rinsed with PBS and 5 random images were taken per matrix using an Olympus IX80 laser scanning confocal microscope, as previously described [17]. 

### Subcutaneous Implant Study

CD-1 male nude mice (6-8 wk-old; Charles River Laboratories) were subjected to a single tail vein injection of STZ (220mg/kg of body weight) or vehicle control (non-diabetic mice, sodium citrate buffer) to generate our model of T1D [18]. Blood glucose levels of fasting animals (4h fast) were measured 7-10d post-injection to confirm hyperglycemia. Average fasting blood glucose values were 15.1±0.8mM for diabetic mice and 4.8±0.4mM for non-diabetic controls (*p*<0.0001). Four weeks post-STZ injection (or vehicle control), the mice were anaesthetized with isoflurane and each mouse received 4 dorsal subcutaneous hydrogel implants consisting of: 1) collagen matrix; 2) collagen-chitosan matrix; 3) collagen matrix+CAC; and 4) collagen-chitosan matrix+CAC. After 1, 2 or 6 weeks, mice were sacrificed and the implants were removed. 

### Histology and Immunofluorescence

Hydrogels were explanted and 1/2 of each were fixed and dehydrated, embedded in OCT, snap frozen with liquid nitrogen and kept at -80°C. Sections were prepared for hematoxylin phloxine saffron (HPS) staining or immunohistochemistry. For immunofluorescence, sections were incubated with antibodies against human/mouse CXCR4 (1:50; Abcam) for angiogenic cells or human/mouse vWF (1:50, Abcam) for endothelial cells, followed by appropriate secondary antibodies. Slides were imaged using a Zeiss fluorescence microscope. See Supplemental Methods (in File S1) for details. 

### Cytokine Array

Cytokine antibody array (RayBiotech, Inc; Cat#AAM-CYT-G3-8) analysis was performed according to the manufacturer’s protocol using lysates of hydrogel implants (1/2 of each). Expression was first normalized to the amount of protein loaded in the array, and then presented as a fold-change relative to the intensity level for each cytokine in the collagen implant at the given time-point. To determine an overall trend for the expression changes, the sum of the fold-changes for each cytokine (within each of the 3 groups of cytokines) was averaged.

### Statistical Analysis

The data were analyzed using a two or three way ANOVA (linear model) using SAS® version 9.2 (SAS Institute, Cary, NC, USA) and comparisons between individual groups were performed with a Student’s t-test. For mechanical testing data, a Student’s t-test was used to compare each material *P* values < 0.05 were considered significant. *N* values are ≥ 3, with individual *n* values provided in the figure legends.

## Results

### Morphology of the Hydrogels

**Figure 1:**
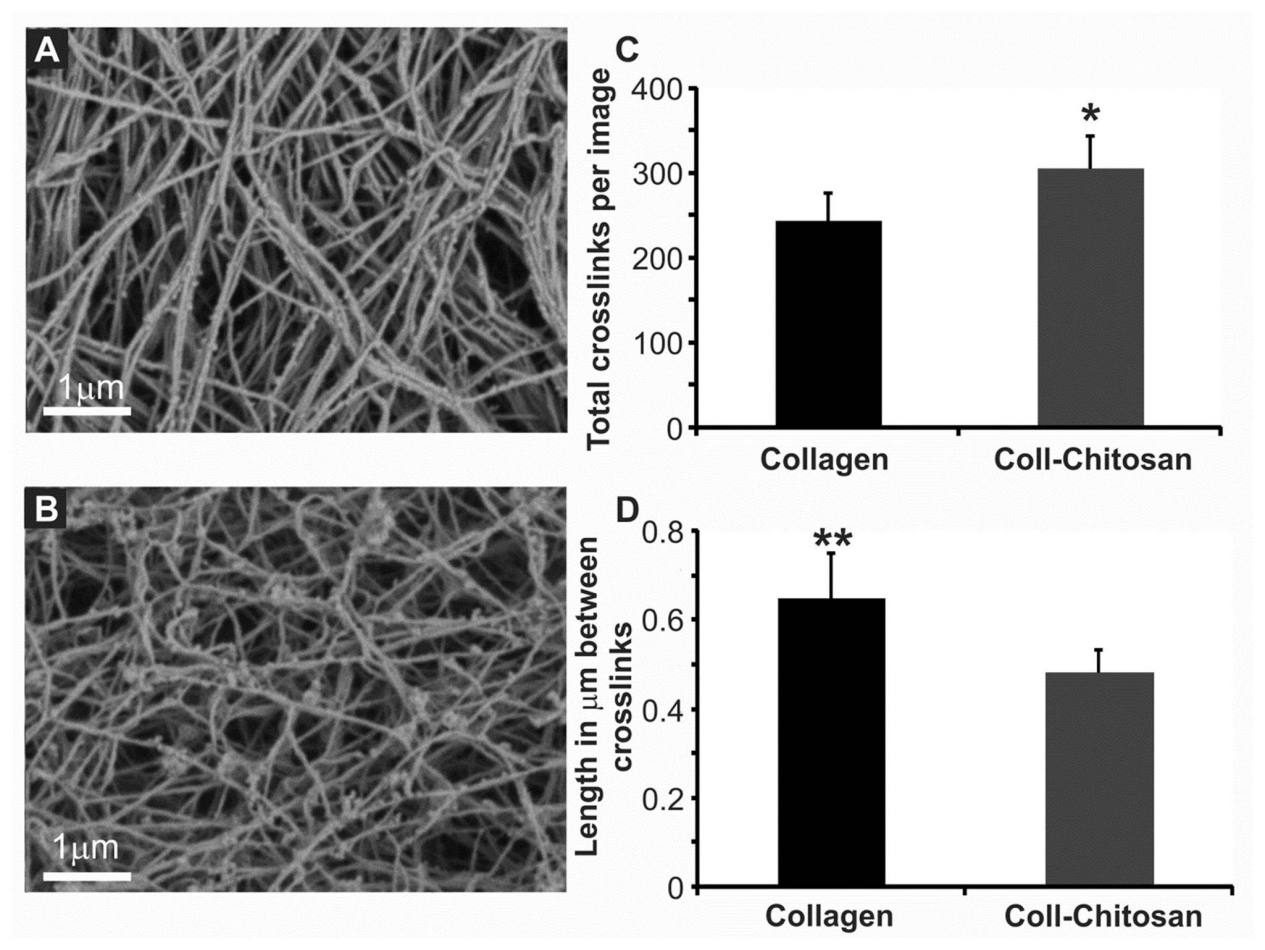
Characterization of hydrogel fiber cross-linking. Representative SEM images of collagen (**A**) or collagen (coll)-chitosan (**B**) hydrogels. Total cross-links per image (**C**) as well as distance (µm) between cross-links (**D**) were quantified (*p=0.046; **p=0.03; *n*=4 each).

Figure 1 shows representative SEM images of the collagen (Figure 1A) and collagen-chitosan (Figure 1B) hydrogels. Fiber diameter was not significantly different between hydrogels (78.6±9.0μm vs. 73.3±8.0μm). However, collagen-chitosan hydrogels had a greater number of cross-links per field-of-view (FOV; Figure 1C, *p*<0.05) and a shorter length between cross-links, compared to collagen hydrogels (Figure 1D, *p*=0.03). 

### Degradation Properties of the Hydrogels

**Figure 2:**
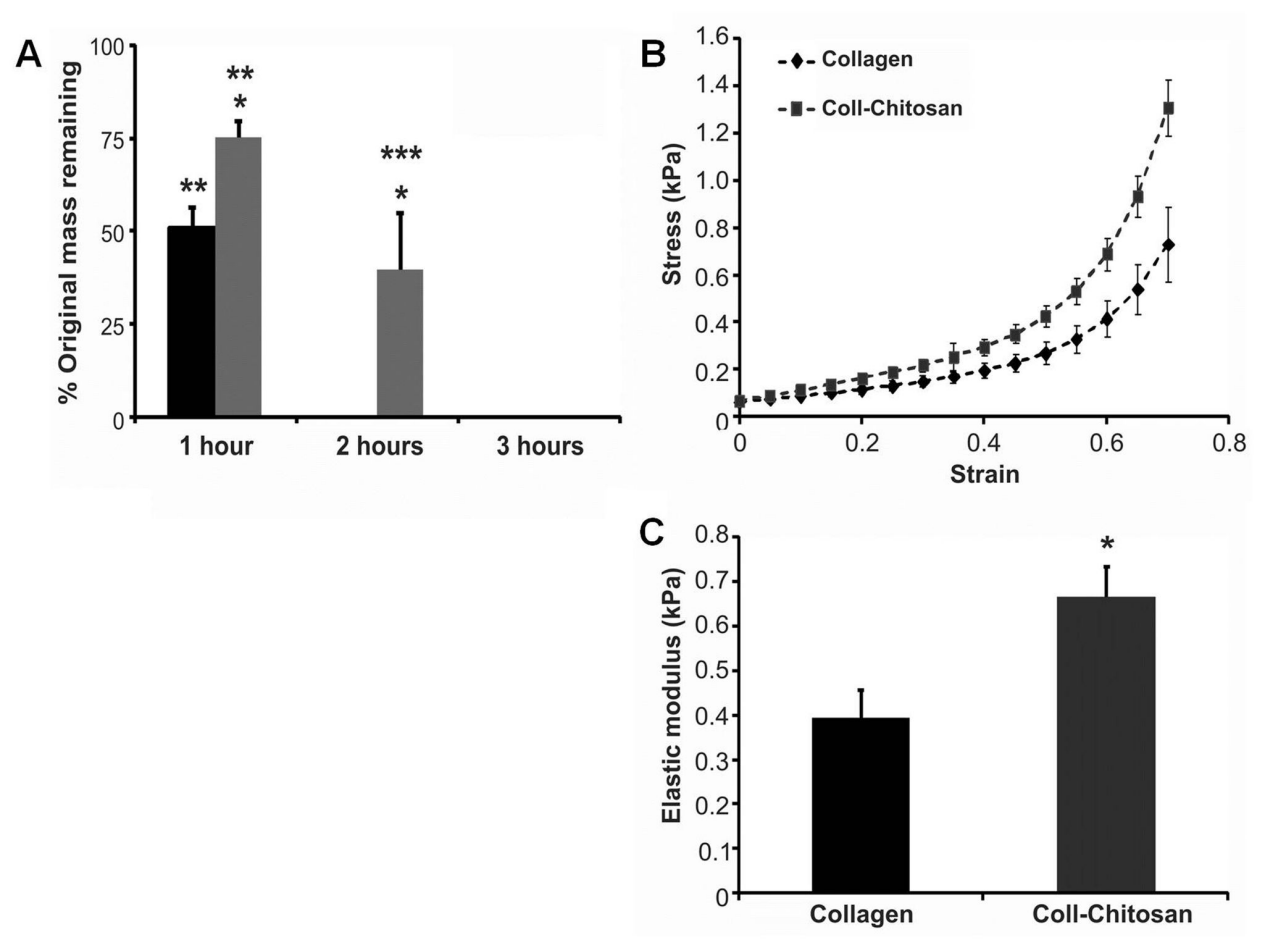
Degradation and elastic modulus of hydrogels. (**A**) Collagen (black bars) and collagen-chitosan (gray bars) hydrogels were incubated in 100U collagenase and the residual mass was determined over time (*p≤0.03 vs. collagen at the same time-point; **p<0.0001 vs. collagen at 2 hours; ***p<0.0001 vs. collagen-chitosan at 3h; *n*=4 each). (**B**) Stress/strain curve for collagen and collagen-chitosan hydrogels. (**C**) The elastic modulus for the collagen and collagen-chitosan hydrogel samples (*p<0.0001; *n*=8).

*In vitro* degradation studies showed that both hydrogels maintained ~80% of their initial mass after 6 weeks in α-amylase (data not shown). In 400 units/ml of collagenase, both hydrogels were completely degraded within 1h (data not shown). When incubated in 100 units/ml of collagenase, the collagen gel degraded more quickly than the collagen-chitosan hydrogels (Figure 2A) after 1h (*p*=0.03) and 2h (*p*<0.0001). Collagenase at 1 unit/ml completely degraded both hydrogels within 24h (data not shown). At a concentration of 0.1 units/ml of collagenase, collagen and collagen-chitosan hydrogels degraded at a similar rate (Table 1). With the exception of 2 weeks, no difference in mass loss was observed between the hydrogels. Mass loss peaked after 4 weeks (Table 1); some of the collagen hydrogels were completely degraded at this time point, but not the collagen-chitosan hydrogels. 

**Table 1:** Long-term *in vitro* collagenase-mediated hydrogel degradation (at 0.1 units/ml of collagenase).

	**Collagen Collagen-Chitosan**	
**Timepoint**	**Mass remaining (%±SE)**	**Mass remaining (%±SE)**
**1 week**	60.5±4.4*	52.9±5.6**
**2 weeks**	49.6±3.6^†^***	33.8±2.6****
**3 weeks**	26.5±2.6	20.0±2.6
**4 weeks**	8.4±3.6	8.9±0.9
**5 weeks**	11.4±2.6	4.9±1.4
**6 weeks**	6.6±4.7	6.7±2.0

Samples at weeks 1-6 were significantly degraded compared to week 0 (*p*<0.0001). *p<0.0001 vs. collagen at 3, 4, 5, and 6 weeks. **p≤0.0008 vs. collagen-chitosan at all other time points. ***p<0.0001 vs. collagen at 3, 4, 5, and 6 weeks. ****p≤0.03 vs. collagen-chitosan at 3, 4, 5 and 6 weeks. ^†^ *p*=0.002 vs. collagen-chitosan at the same time-point.

### Mechanical Characterization of the Hydrogels

Unconfined compression testing revealed that both hydrogels displayed a gradual increase in stiffness and typically showed a linear stress-strain relationship up to 40% strain followed by a non-linear response (Figure 2B). The collagen-chitosan hydrogels showed a statistically significant increase in elastic modulus compared to the collagen hydrogels (Figure 2C; 0.7kPa vs. 0.4kPa at 30% strain, respectively, (*p*<0.0001)).

### Viability of CACs Cultured in Hydrogels

**Figure 3:**
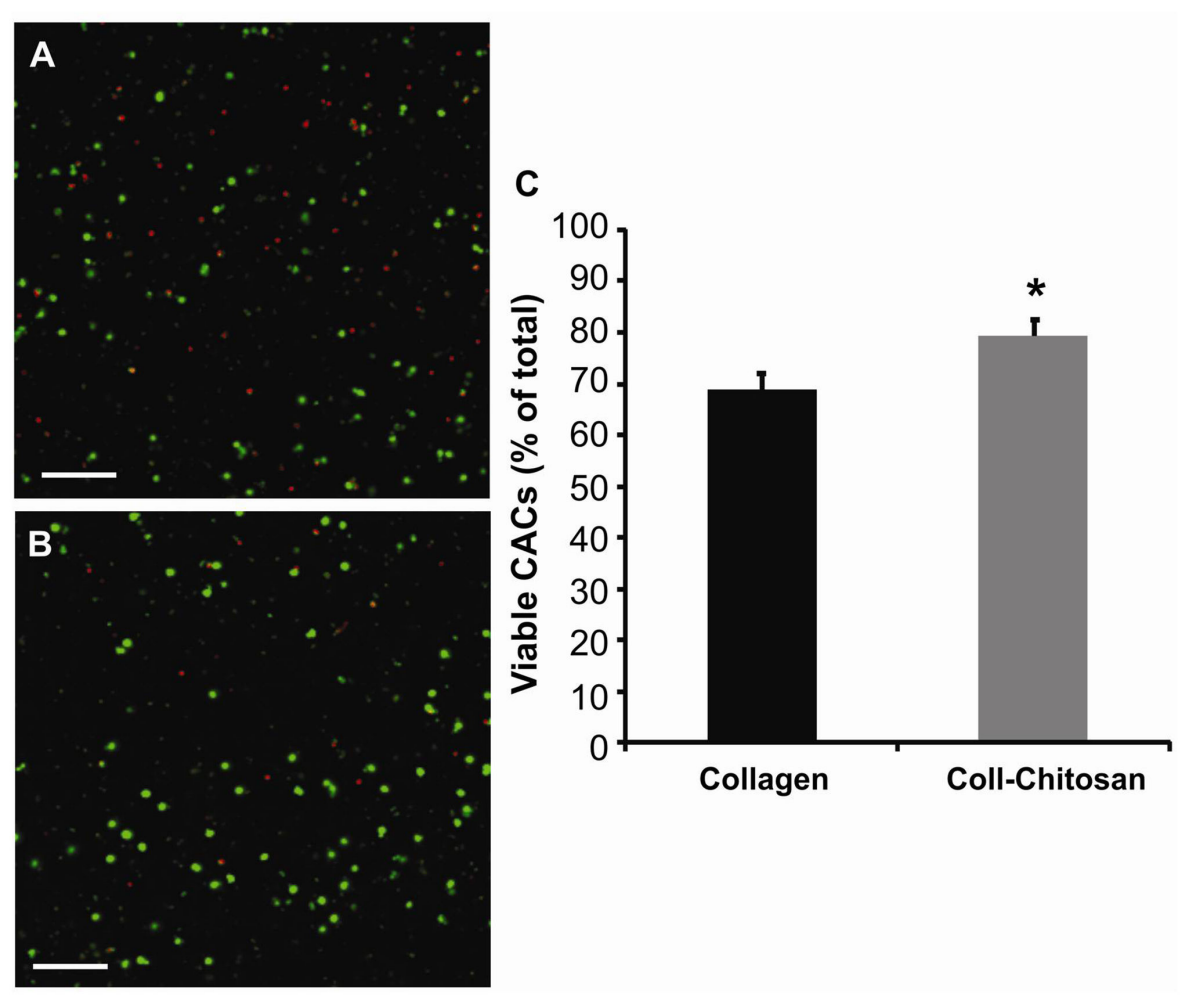
Viability of human CACs on collagen and collagen-chitosan matrices. Representative live/dead images of CACs embedded in collagen (**A**) or collagen-chitosan (**B**) matrices. Scale bars=100µm. (**C**) Graph showing the percentage of viable CACs (live cells/total CAC count; *p=0.01; *n*=3 each).

Both hydrogels promoted high levels of CAC viability after 24h of culture (Figure 3); however there was a significantly higher percentage of viable CACs embedded in the collagen-chitosan hydrogel compared to the collagen hydrogel (79.4±2.7% vs. 68.9±3.2%, *p*=0.01). 

### *In Vivo* Cell Invasion in Subcutaneous Hydrogel Implants

**Figure 4:**
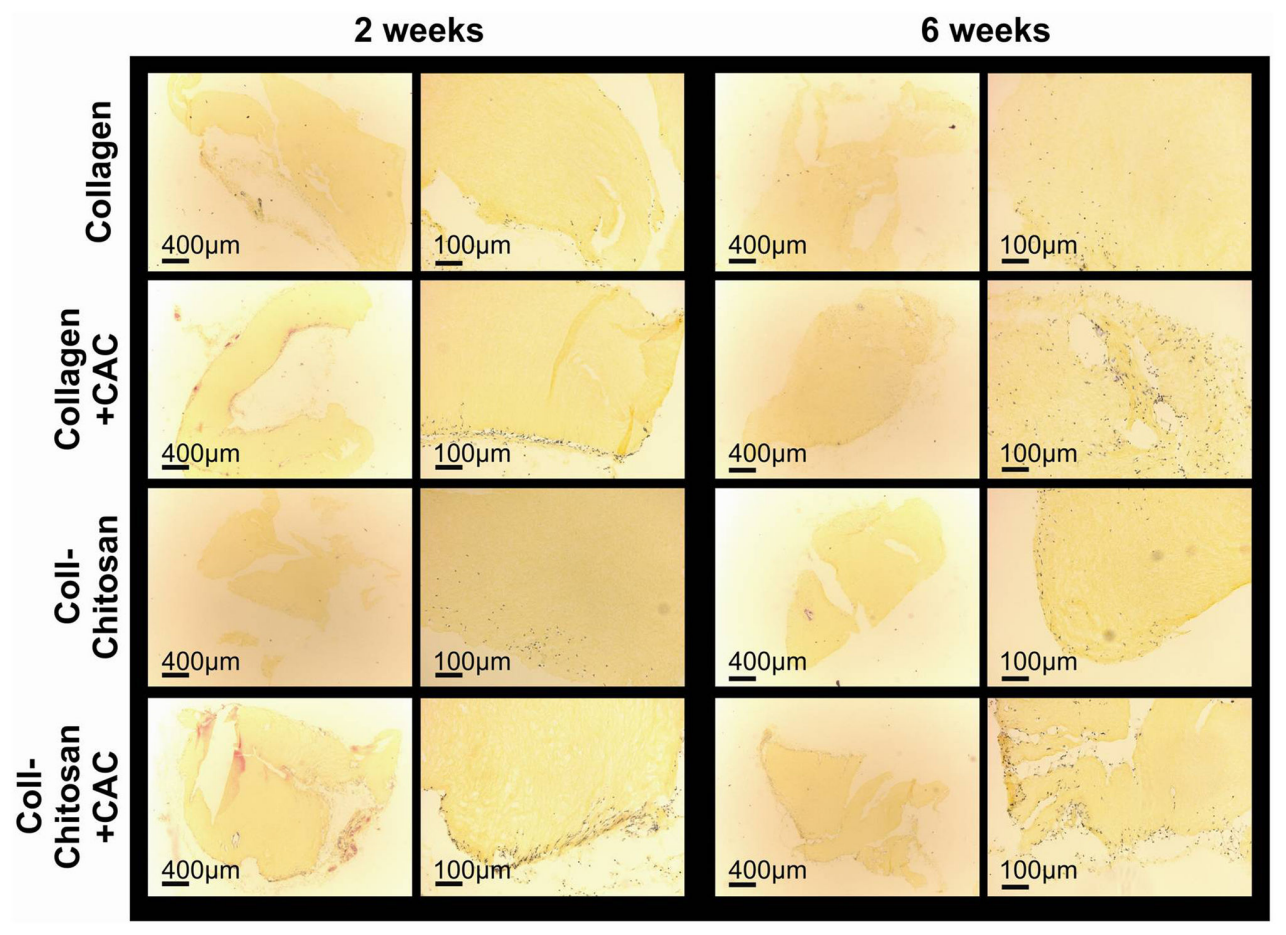
Representative images of HPS-stained collagen and collagen-chitosan hydrogels (±CACs) explanted at 2 and 6 weeks.

Collagen and collagen-chitosan hydrogels ± human CACs were subcutaneously implanted into 4 week diabetic nude mice for a period of 1, 2 or 6 weeks. At 1 week, host cells had infiltrated the collagen and collagen-chitosan hydrogels to a similar extent (Figure S1 in File S1). By 6 weeks the hydrogels with CACs appeared to have a greater number and distribution of infiltrated cells as observed qualitatively in the HPS-stained sections (Figure 4). There was a greater number of CXCR4^+^ cells in the collagen+CAC implants at 6 weeks versus 2 weeks (*p*=0.03; Figure S2 in File S1). Overall, no difference in the number of vWF^+^ (endothelial cell marker) and CXCR4^+^ (angiogenic cell marker) cells was seen between the 4 implant types at 2 and 6 weeks (Figure S2 in File S1). These results are in contrast to our previous work in a non-diabetic model comparing collagen vs. collagen-chitosan hydrogels, which demonstrated increased vascular/angiogenic cell invasion with the addition of chitosan to the collagen hydrogel [14,19]. To better understand this difference, cytokine expression changes were evaluated in hydrogels implanted subcutaneously in diabetic and non-diabetic mice.

### Cytokine Expression Profiles of Hydrogel Implants

Cytokine expression was evaluated in matrix implants at baseline (1 week), 2 weeks and 6 weeks. The cytokines have been grouped into 3 categories: pro-angiogenic/pro-islet, pro-angiogenic/anti-islet, and anti-angiogenic/anti-islet proteins (Table S1 in File S1). Tables S2 through S7 (in File S1) provide the relative intensity level/mg protein for the cytokines in these categories for the collagen matrix implants, to which levels in the other matrix groups is compared. Since each mouse serves as its own control (each animal’s matrix implant data is normalized to its own collagen matrix implant), we do not directly compare the non-diabetic versus diabetic results. In collagen matrix implants in diabetic mice, the pro-angiogenic/pro-islet proteins GM-CSF and VCAM-1 increased over time, while SCF, SDF-1, and VEGF were highest at 1 week and then decreased with time (Table S2 in File S1). Levels of the pro-angiogenic/anti-islet proteins, IL-1β, MCP-5, MIP-1γ, MIP-3α, RANTES, and TNF-α decreased or remained unchanged over time, while lymphotactin, MCP-1, M-CSF and TARC increased compared to the 1 week levels (Table S3 in File S1). The pro-inflammatory, anti-angiogenic/anti-islet proteins showed varying trends, with BLC decreasing over time, IFN-γ and PF-4 peaking at 2 weeks and then decreasing, and IL-12p70 and MIG increasing over the 6-week period (Table S4 in File S1).

In non-diabetic mice, the collagen matrix had higher levels of pro-angiogenic/pro-islet cytokines GM-CSF, SCF, SDF-1α and VCAM-1 at 2 and 6 weeks compared to 1 week, whereas VEGF levels decreased with time (Table S5 in File S1). Levels of the pro-angiogenic/anti-islet proteins IL-1β, lymphotactin, M-CSF, and TARC were higher, whereas MCP-1, MCP-5, RANTES, and TNF-α levels were lower at 6 weeks compared to the 1-week time point (Table S6 in File S1). Similar to the diabetic mice, the anti-angiogenic/anti-islet proteins showed varying trends, with BLC and PF-4 decreasing over time, IFN-γ peaking at 2 weeks and then decreasing, and IL-12p70 and MIG increasing over the 6-week period (Table S7 in File S1).

**Figure 5:**
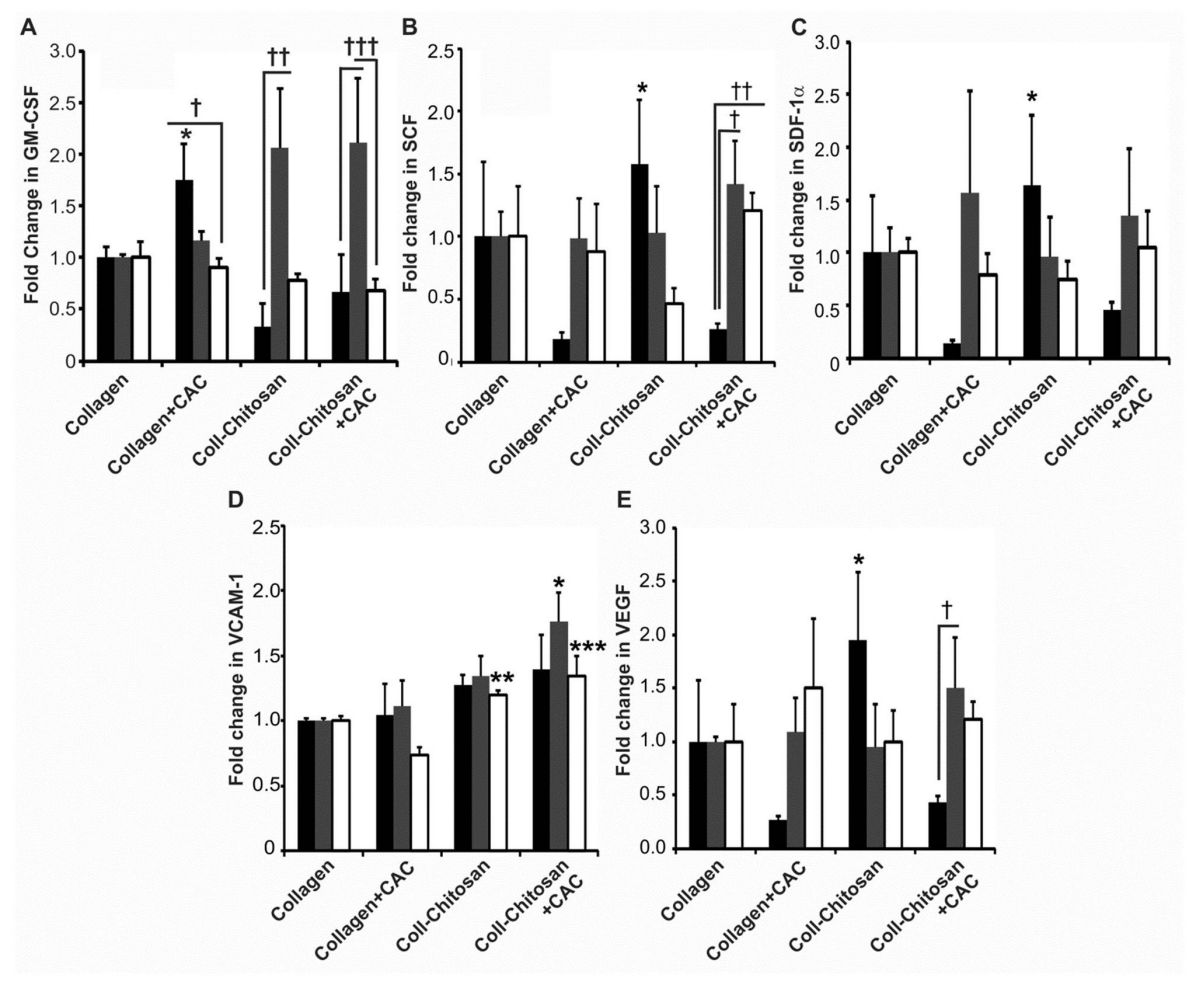
Expression of pro-angiogenic/pro-islet cytokines in subcutaneous implants in diabetic mice. The expression of GM-CSF (**A**), SCF (**B**), SDF-1α (**C**), VCAM-1 (**D**) and VEGF (**E**) protein in hydrogels explanted at 1 (black bars), 2 (grey bars) and 6 weeks (white bars) was normalized to the levels in the collagen hydrogel at their respective time point (*n*=3 each). *P*-values in (A): *p=0.007 and *p*=0.04 vs. 1 week collagen-chitosan and collagen-chitosan+CAC implants, respectively; ^**†**^ *p*=0.02; ^**††**^ *p*=0.005; and ^**†††**^ *p*<0.05). In (B): *p≤0.004 vs. collagen+CAC and collagen-chitosan+CAC implants at 1 week; ^**†**^ *p*=0.002; and ^**††**^ *p*=0.01. In (C): *p=0.01 vs. collagen+CAC at 1 week. In (D): *p=0.01 vs. collagen at 2 weeks; **p=0.004 vs. collagen+CAC at 6 weeks; ***p=0.0002 vs. collagen+CAC at 6 weeks. In (E): *p≤0.007 vs. collagen+CAC and collagen-chitosan+CAC at 1 week; and ^**†**^ *p*=0.03.

**Figure 6:**
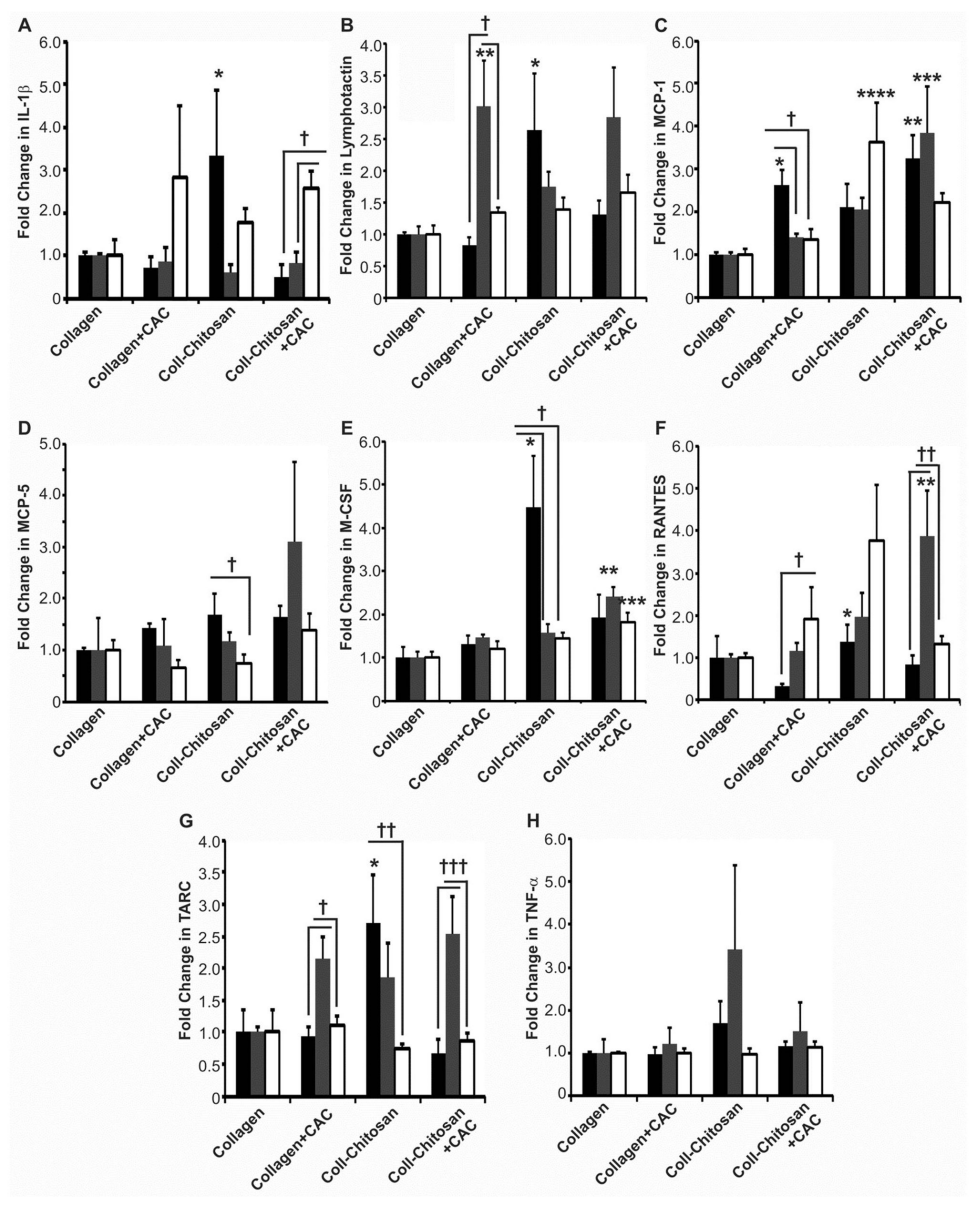
Expression of pro-angiogenic/anti-islet cytokines in subcutaneous implants in diabetic mice. The expression of IL-1β (**A**), lymphotactin (**B**), MCP-1 (**C**), MCP-5 (**D**), M-CSF (**E**), RANTES (**F**), TARC (**G**) and TNF-α (**H**) protein in hydrogels explanted at 1 (black bars), 2 (grey bars) and 6 weeks (white bars) was normalized to the levels in the collagen hydrogel at their respective time point (*n*=3 each). *P*-values in (A): *p=0.05 vs. collagen-chitosan+CAC at 1 week; and ^**†**^ *p*≤0.003. In (B): *p=0.03 vs. collagen+CAC at 1 week; **p=0.04 vs. collagen at 2 weeks; and ^**†**^ *p*≤0.02. In (C): *p=0.04 vs. collagen at 1 week; **p=0.003 vs. collagen at 1 week; ***p≤0.01 vs. collagen and collagen+CAC hydrogels at 2 weeks; ****p=0.01 vs. collagen and collagen+CAC at 6 weeks; and ^**†**^ *p*≤0.006 vs. collagen+CAC at 2 and 6 weeks. In (D): ^**†**^ *p*=0.04. In (E): *p≤0.04 vs. all other hydrogels at 1 week; **p≤0.003 vs. all other hydrogels at 2 weeks; ***p=0.02 vs. collagen at 6 weeks; and ^**†**^ *p*≤0.02. In (F): *p=0.009 vs. collagen+CAC at 1 week; **p≤0.01 vs. collagen and collagen+CAC at 2 weeks; ^**†**^ *p*=0.04; and ^**††**^ *p*≤0.02. In (G): *p≤0.02 vs. all other implants at 1 week; ^**†**^ *p*≤0.007; ^**††**^ *p*=0.03; and ^**†††**^ *p*≤0.008.

**Figure 7:**
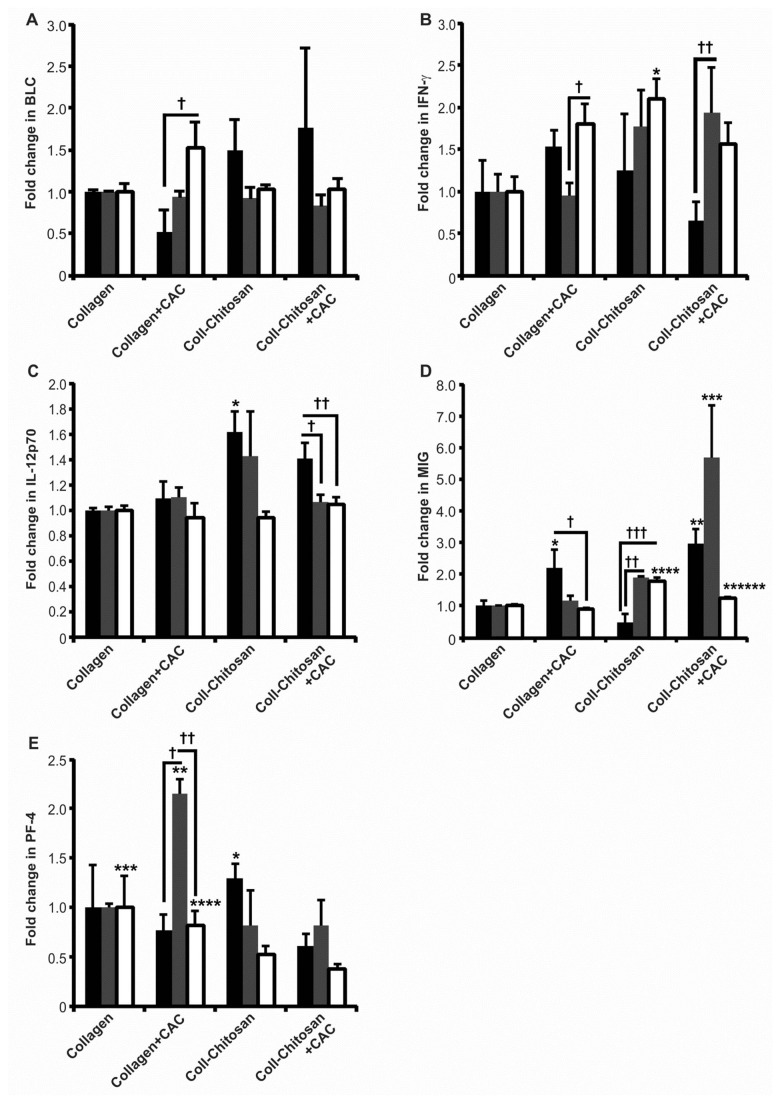
Expression of anti-angiogenic/anti-islet cytokines in subcutaneous implants in diabetic mice. The expression of BLC (**A**), IFN-λ (**B**), IL12p70 (**C**), MIG (**D**), and PF-4 (**E**) protein in hydrogels explanted at 1 (black bars), 2 (grey bars) and 6 weeks (white bars) was normalized to the levels in the collagen hydrogel at their respective time point (*n*=3 each). *P*-values in (A): ^**†**^ *p*=0.02. In (B): *p=0.02 vs. collagen at 6 weeks; ^**†**^ *p*=0.01; and ^**††**^ *p*=0.04. In (C): *p≤0.02 vs. collagen and collagen+CAC implants at 1 week; ^**†**^ *p*=0.02; and ^**††**^ *p*=0.02). In (D): *p=0.02 vs. collagen-chitosan at 1 week; **p≤0.02 vs. collagen and collagen-chitosan at 1 week; ***p≤0.01 vs. all other implants at 2 weeks; ****p≤0.001 vs. all other implants at 6 weeks; *****p=0.03 vs. collagen+CAC at 6 weeks; ^**†**^ *p*=0.02; ^**††**^ *p*<0.0001; and ^**†††**^ *p*=0.009. In (E): *p≤0.03 vs. collagen-chitosan+CAC and collagen+CAC implants at 1 week; **p≤0.006 vs. all other implants at 2 weeks; ***p≤0.01 vs. collagen-chitosan and collagen-chitosan+CAC at 6 weeks; ****p=0.007 vs. collagen-chitosan+CAC at 6 weeks; ^**†**^ *p*<0.0001; and ^**††**^ *p*<0.0001.

For the expression profiles (Figures 5-7; Figures S3-S5 in File S1), each mouse had the cytokine values for the 4 implant types normalized to its own collagen matrix implant (each mouse received one of each implant type) to minimize the effects of inter-mouse variability.

### Expression Profiles of Pro-Angiogenic/Pro-Islet Proteins

Figure 5 shows the expression of pro-angiogenic/pro-islet cytokines in diabetic mice. Collagen+CAC implants at 1 week had significantly higher levels of GM-CSF than either collagen-chitosan implants (*p*=0.007) or collagen-chitosan+CAC implants (*p*=0.04; Figure 5A). The level of GM-CSF in collagen+CAC implants decreased from 1 to 6 weeks (*p*=0.02; Figure 5A). GM-CSF expression was greatest in chitosan-containing hydrogels (± CACs) at 2 weeks (*p*<0.05 versus 1 and 6 weeks; Figure 5A). At 1 week, the expression of SCF (Figure 5B) and/or SDF-1α (Figure 5C) was higher in the collagen-chitosan matrix compared to collagen+CAC (SCF, *p*=0.003) and collagen-chitosan+CAC matrices (SCF, *p*=0.004 and SDF-1α, *p*=0.01). At 2 weeks, VCAM-1 was higher in collagen-chitosan+CAC versus collagen hydrogels (Figure 5D; *p*=0.01), and at 6 weeks its level was greater in collagen-chitosan (*p*=0.004) and collagen-chitosan+CAC (*p*=0.0002) implants compared to collagen+CACs (Figure 5D). VEGF was increased in the collagen-chitosan hydrogel at week 1 (compared to collagen+CAC (*p*=0.003) and collagen-chitosan+CAC hydrogels (*p*=0.007)) and was higher in the collagen-chitosan+CAC hydrogel at 2 weeks compared to 1 week (*p*=0.03; Figure 5E). 

For comparison, (Figure S3 in File S1) provides results for the levels of pro-angiogenic/pro-islet cytokines in non-diabetic mice. GM-CSF levels were higher in the collagen-chitosan (2 weeks) and collagen-chitosan+CAC (2 and 6 weeks) hydrogels versus the collagen hydrogels (*p*≤0.03; Figure S3A in File S1). SCF levels were lower at 1 week in collagen-chitosan+CAC hydrogels compared to collagen (*p*=0.02; Figure S3B in File S1). Also, collagen-chitosan+CAC hydrogels had higher levels of SDF-1α, VCAM-1 and VEGF at 6 weeks compared to collagen (*p*≤0.04; Figures S3C-E in File S1).

### Expression Profiles of Pro-Angiogenic/Anti-Islet Proteins

Figure 6 shows the profile of cytokines known to be pro-angiogenic, but also with evidence for roles in decreasing islet survival, or in promoting T-cell activation and a prolonged pro-inflammatory response, which could lead to graft rejection (see Table S1 in File S1). There was a significant increase in IL-1β at 1 week for the collagen-chitosan implant versus the other matrices (*p*≤0.05; Figure 6A). In collagen-chitosan+CAC implants, IL-1β increased at 6 weeks compared to its earlier time points (*p*=0.0007 and *p*=0.003 for 1 and 2 weeks, respectively; Figure 6A). The addition of chitosan or CACs resulted in an increase in lymphotactin compared to the collagen hydrogel at various time points (Figure 6B). MCP-1 was increased with the addition of CACs to collagen (at 1 week; *p*=0.03) and to collagen-chitosan hydrogels (at 1 and 2 weeks; *p*=0.003) compared to collagen implants (Figure 6C). At 6 weeks, the collagen-chitosan hydrogels had greater MCP-1 expression compared to collagen ± CAC implants (*p*=0.01; Figure 6C). MCP-5 expression was greater at 1 week versus the 6 week time-point for collagen-chitosan implants (Figure 6D, *p*=0.04). The M-CSF level was greater in collagen-chitosan implants compared to all other groups at 1 week (*p*<0.05), but decreased at 2 and 6 weeks compared to its baseline level (*p*=0.02 and *p*=0.01, respectively; Figure 6E). In addition, the collagen-chitosan+CAC implants had higher M-CSF than the other conditions at 2 weeks (0.003<*p*<0.0001), and higher than collagen at 6 weeks (*p*=0.02; Figure 6E). RANTES expression was higher for collagen-chitosan versus collagen+CAC implants at 1 week (*p*=0.009), as well as for collagen-chitosan+CAC versus collagen ± CAC implants at 2 weeks (*p*=0.01; Figure 6F). At 1 week, TARC was more abundant in collagen-chitosan implants compared to all other groups (*p*≤0.02; Figure 6G), but no differences were observed between groups at 6 weeks. Although there was a trend for increased TNF-α expression at 1 and 2 weeks for the collagen-chitosan group, no significant differences were observed (Figure 6H). 

For comparison, (Figure S4 in File S1) provides results for the levels of pro-angiogenic/anti-islet cytokines in the 4 implant types in non-diabetic mice. To summarize, collagen-chitosan+CAC hydrogels had higher IL-1β levels at 1 and 2 weeks versus collagen hydrogels (*p*≤0.02; Figure S4A in File S1). Lymphotactin levels were increased in the collagen-chitosan (1 week) and collagen-chitosan+CAC (1 and 6 weeks) hydrogels compared to collagen (*p*≤0.04; Figure S4B in File S1). MCP-1 levels were higher at 6 weeks in collagen-chitosan and collagen-chitosan+CAC hydrogels versus collagen hydrogels (Figure S4C in File S1). Compared to collagen hydrogels, MCP-5 levels were elevated at 2 weeks for collagen-chitosan and at all time-points for collagen-chitosan+CAC hydrogels (*p*≤0.02; Figure S4D in File S1). At various time points, M-CSF, RANTES and TARC levels were higher in the collagen-chitosan and collagen-chitosan+CAC hydrogels compared to their respective collagen hydrogel controls (*p*≤0.047; Figures S4E-G in File S1). TNF-α levels were higher in collagen-chitosan (6 weeks) and collagen-chitosan+CAC (1 and 6 weeks) versus collagen hydrogels (*p*≤0.049; Figure S4H in File S1).

### Expression Profiles of Anti-Angiogenic/Anti-Islet Proteins

The expression of anti-angiogenic/anti-islet proteins in implants at 1, 2 and 6 weeks is presented in Figure 7. No difference in BLC levels were seen between the implant types; however it did increase in the collagen+CAC group between 1 and 6 weeks (*p*=0.02; Figure 7A). IFN-γ levels tended to increase for all groups compared to collagen implants. At 6 weeks, IFN-γ expression was higher for collagen-chitosan versus collagen implants (Figure 7B, *p*=0.02). IL-12p70 was increased at 1 week for collagen-chitosan versus both collagen (*p*=0.005) and collagen+CAC implants (*p*≤0.02; Figure 7C). MIG expression was greater in implants containing cells, compared to the collagen-chitosan implant at 1 week (*p*≤0.02; Figure 7D). At 2 weeks, MIG was increased in the collagen-chitosan+CAC implants versus all other groups (*p*≤0.01; Figure 7D), and at 6 weeks, levels were higher in the collagen-chitosan implant versus all other groups (*p*≤0.001; Figure 7D). Collagen+CAC implants showed highest expression at 1 week (Figure 7D), significantly higher than collagen-chitosan hydrogels (*p*=0.02). The addition of chitosan decreased the 6 week expression of PF-4 compared to collagen implants (*p*=0.01); while expression in the collagen-chitosan+CAC implants was reduced compared to the collagen+CAC group, also at 6 weeks (*p*=0.0009; Figure 7E). 

For comparison, (Figure S5 in File S1) provides results for the levels of anti-angiogenic/anti-islet cytokines in non-diabetic mice. Briefly, 2-week levels of BLC were higher in collagen-chitosan±CAC hydrogels compared to collagen (*p*≤0.03; Figure S5A in File S1). IFN-γ and IL-12p70 was increased in the collagen-chitosan+CAC hydrogels at 1 week compared to collagen, but this difference was lost at later time-points (*p*≤0.04; Figures S5B, C in File S1). At various time points, MIG levels were increased in all hydrogel types compared to the collagen hydrogels (*p*≤0.04; Figure S5D in File S1). At 6 weeks, PF-4 levels were reduced in collagen-chitosan±CAC hydrogels versus collagen (*p*=0.04; Figure S5E in File S1).

### Summary of Changes in Cytokine/Protein Expression

**Figure 8:**
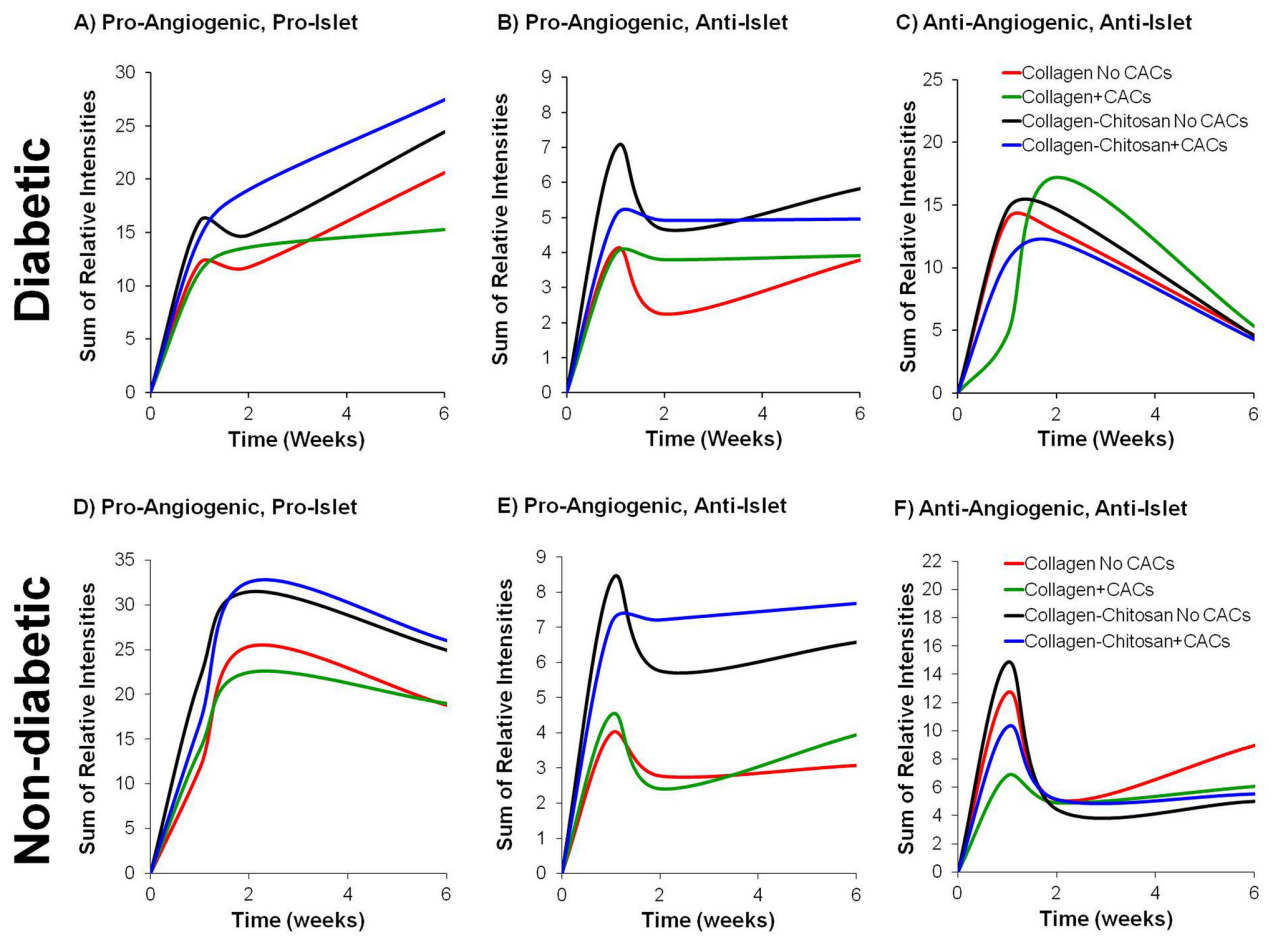
Cytokine profile summary for implants in diabetic and non-diabetic mice. Cytokine profiles are presented for the three groups of cytokines: pro-angiogenic/pro-islet, pro-angiogenic/anti-islet, and anti-angiogenic/anti-islet for implants in diabetic (A-C) and non-diabetic (D-F) mice. Each implant type is graphed as a separate colour: collagen (red); collagen+CACs (green); collagen-chitosan (black); and collagen-chitosan+CACs (blue). Relative expression intensities at given time points post-implantation were normalized per mg of protein for each sample, and then summated for each group of cytokines.

Figure 8 is a graphic summary of the expression changes over time for the three cytokine/protein groups evaluated: pro-angiogenic/pro-islet; pro-angiogenic/anti-islet; and anti-angiogenic/anti-islet. The expression pattern in diabetic mice suggests that the addition of chitosan or chitosan+CACs can help stimulate the production of pro-angiogenic/pro-islet cytokines, for which levels continue to increase over the 6-week period (Figure 8A, B). For all implants in diabetics, the expression of the anti-angiogenic/anti-islet cytokines peaks at 1-2 weeks, and then undergoes up to a ~3-fold reduction by 6 weeks (Figure 8C). In comparison, the patterns suggest that the rate of increase and the magnitude of pro-angiogenic/pro-islet cytokine expression are greater in non-diabetic mice than in diabetics; with the collagen-chitosan and collagen-chitosan+CAC implants exhibiting the greatest expression levels (Figure 8D, E). Furthermore, resolution of the peak in anti-angiogenic/anti-islet cytokines appears to occur sooner for all matrix implant types in non-diabetic (Figure 8F) versus diabetic mice.

## Discussion

Many factors need to be considered in developing an ectopic islet transplant site, including islet retention, survival, and function, as well as physical properties of the biomaterial implant and its vascularization. This study focused on preparing a biomaterial with suitable physical characteristics and the ability to promote a pro-angiogenic environment at the implant site. We demonstrated that the addition of chitosan to a collagen-based hydrogel increased its cross-linking density, its mechanical strength and its ability to support the viability of angiogenic cells. Previously, we showed that adding chitosan to a collagen hydrogel could increase recruitment of vWF^+^ and CXCR4^+^ cells and improve blood vessel growth in a non-diabetic mouse model [14,19], but the same was not observed in the present study using a T1D model. This may be explained, in part, by the observation that the increase in pro-angiogenic cytokine levels in matrix implants in diabetic mice was less in magnitude and took longer to occur, compared to the non-diabetic groups. Notably, this study also showed that the cytokine profile generated in response to implantation of the transplant site may be an important consideration in determining the optimal timing for promoting a pro-angiogenic environment and while reducing levels of anti-islet cytokines. Overall, this data suggests that using a collagen hydrogel with chitosan and CACs may be a good strategy for preparing a pro-angiogenic ectopic site for islet transplantation. 

To characterize the cytokine response, the different proteins being assayed were categorized by their pro-angiogenic, pro-islet, anti-angiogenic and/or anti-islet functions, based on the literature (Table S1 in File S1). As shown in the graphic summaries (Figure 8), the addition of chitosan or CACs to the collagen implants helped to stimulate a more pro-angiogenic environment without stimulating long-term anti-islet protein production. The levels of anti-angiogenic/anti-islet cytokines peaked 1 to 2 weeks after implantation in diabetic mice, and then declined up to the 6 week time-point. The expression of pro-angiogenic/pro-islet cytokines was comparable to the anti-angiogenic/anti-islet cytokines for up to 2 weeks, but then increased up to 6 weeks post-implantation. For our matrices, this suggests that the optimal time for transplantation of islets into the ectopic site is likely to be approximately 2 weeks post-implantation. At this time, a favorable cytokine environment has been generated – inhibitory cytokine levels are in decline, while pro-angiogenic/pro-islet cytokines are increasing. 

Greater expression of several pro-angiogenic cytokines was observed in implants with chitosan or CACs in diabetic mice at 1 week. The increase in VCAM-1 at 6 weeks in the collagen-chitosan hydrogels (±CACs) suggests an increase in endothelial phenotype cells in these matrices. Other than for VCAM-1, the significantly different comparison groups varied for the different angiogenic cytokines and a superior implant type could not be clearly identified based on individual cytokine analysis; however, the sum expression profiles suggest that the addition of chitosan±CACs generates a more pro-angiogenic implant milieu in the T1D model. Not all cytokines with pro-angiogenic functions may be beneficial over the long-term, since many of these can also have a negative influence on islet survival, such as IL-1β, TNF-α, IFN-γ, monocyte chemokines, and T-lymphocyte chemokines/activation proteins (see Table S1 in File S1). Exposure of islets to such cytokines can increase apoptosis and decrease glucose responsiveness [20,21] and can lead to a pro-inflammatory state that recruits more inflammatory cells [22,23]. Our matrices were able to stabilize the levels of pro-angiogenic/anti-islet cytokines over time, while the pro-angiogenic/pro-islet cytokines increased from 2-6 weeks post-implantation; suggesting that the balance shifted towards a pro-islet environment, which was most prominent in the groups with chitosan. Based on the overall cytokine analysis, it appears that the collagen-chitosan matrix (± CACs) may be the best condition to achieve the pro-angiogenic, pro-islet environment needed for islet transplantation. The delivery of islets into these transplant sites remains to be performed in order to determine if the predicted optimal time-point for islet transplantation (2 weeks) is in fact ideal for promoting islet survival and function, and this constitutes a future research direction. 

There have been several attempts to pre-vascularize an ectopic site for islet transplantation using various biomaterials and devices with varying degrees of success [11,24-29]. These studies have tested different pre-vascularization periods (from as little as 1 week, and up to 3 months), and some have implanted the material before rendering the animal diabetic. However, low insulin and uncontrolled glucose levels have been shown to contribute to poor neovascularization [30]; and the survival of islet allografts is decreased in rats with chronic versus acute onset diabetes [31]. Therefore, we believe that use of a chronic diabetes model will best replicate the transplant situation, and should be used to rigorously test strategies for enhancing vascularization in a diabetic milieu. 

As an example of how the model system is critical in assessing the vasculogenic potential of an implant material, we can compare the present work with our previous collagen-chitosan matrix studies. We found that adding chitosan, attractive for use in engineering vascularized tissues (reviewed in 32), to a collagen matrix promoted significant recruitment of angiogenic cells (vWF^+^ and CXCR4^+^ cells) and blood vessel growth *in vitro* and *in vivo* [14,19]; but these results were generated in normoglycemic / non-diabetic conditions. In the present study, there was minimal recruitment of angiogenic cells to the implants in a chronic T1D model. Even with the transplantation of non-diabetic CACs from healthy human volunteers, we did not observe vascularization to the extent seen previously in the non-diabetic mouse [14]. This may be attributed to the fact that paracrine signaling and neovascularization are defective in diabetes [33,34]. Tissues signal for new vasculature growth, in part, through chemokine-induced recruitment of CACs [35,36], which exhibit impaired mobilization and function in T1D [37,38]. In concordance with defective signaling mechanisms and angiogenesis in diabetes, the production of pro-angiogenic cytokines in implants in T1D mice in the present study was reduced and took longer to occur compared to non-diabetic mice. In addition, the peak in anti-angiogenic/anti-islet cytokines occurs sooner and resolves itself more quickly for implants in non-diabetic versus diabetic mice. Therefore, unlike the results demonstrated in the non-diabetic models, it seems that the addition of CACs or of chitosan to the collagen matrix is not sufficient to enhance vascular cell recruitment or ameliorate the implant environment in diabetic conditions. Therefore, in order to achieve adequate vascularization of ectopic islet transplant sites, it may be necessary to concomitantly address the underlying defects in endogenous angiogenic cell populations that limit neovascularization in diabetes. Several strategies to ameliorate the function of vascular/angiogenic cells in diabetes have been previously reviewed [39], which could be combined with ectopic islet transplantation therapy.

The collagen and collagen-chitosan formulations we report here were modified from our previous study [14]. They differ by the addition of cells prior to gelation, and also by a higher concentration of glycine, which was a component of the cell suspension. We previously reported that glycine in the cell suspension is a successful strategy for protecting CACs from unreacted EDC-NHS cross-linker and increasing their viability [17]. Since the addition of cells can alter a material’s properties [17], and the matrix formulations used in the present study were different, we needed to re-evaluate their mechanical properties. This is important because the mechanical properties, and in particular the elasticity, of a cell’s extracellular environment have a key role in regulating cell differentiation and function [40-42].

The addition of chitosan to the collagen hydrogel resulted in hydrogels with greater cross-link density and superior strength, but which were still degradable by physiologically relevant enzymes. The cross-link density has a role in regulating the porosity of a material. Porosity can control cell migration/invasion rates, which is critical for vascularization [43]. In addition, cross-link density and porosity will play a role in controlling the diffusion of glucose and insulin to and from the islet graft [44]. We have previously shown that our collagen and collagen-chitosan matrices support the survival and function of insulin- and glucagon-positive islets [19]; however, the transport properties of the materials, and whether the higher cross-link density of the collagen-chitosan hydrogel affects this, remains to be determined. It is however, also pertinent to consider that the transport of small (insulin) to intermediate (growth factors) size proteins within the islet-hydrogel implant should be ameliorated with its successful vascularization. 

In terms of *in vivo* material durability, we could retrieve most implants at week 6; however some of the collagen hydrogels could not be found or were too small for analysis other than cytokine arrays. This suggests that the addition of either CACs or chitosan helps the collagen hydrogel implant retain its shape and integrity, which is supported by our previous studies [14] and the presently reported in vitro degradation studies. Overall, the mechanical strength of the collagen-chitosan hydrogel was greater than collagen-only. The range of elastic moduli for both matrix formulations (hydrated) was similar to those reported for a poly(ethylene glycol) hydrogel developed for the encapsulation and rapid recovery of viable and functional 3D β-cell spheroids [45], and a gelatin-poly (vinylpyrrolidone) hydrogel which supported mouse islets for up to 30d [46]. Therefore, it appears that our matrices, and in particular the collagen-chitosan hydrogels, have suitable physical properties for the support of angiogenic cells and islets.

## Conclusion

The current study evaluated collagen-based hydrogels as pro-angiogenic environments suitable for islet transplantation in a chronic T1D model system. The addition of chitosan to the collagen matrix increased its cross-link density and mechanical strength, and supported greater viability of encapsulated CACs. We identified that the cytokine milieu generated within the implant may be an important factor in determining the ideal time to create the pro-angiogenic ectopic site, to ensure that anti-islet protein levels will not be inhibitory to the survival and function of subsequently transplanted islets. The addition of chitosan and CACs to the collagen hydrogel stimulates a more pro-angiogenic cytokine profile. However, compared to the non-diabetic model, in T1D these effects are minimal, which likely contributes to the inability of the collagen-chitosan-CAC implant to improve vWF^+^ and CXCR4^+^ vascular/angiogenic cell recruitment. Together this data suggests that using a collagen hydrogel with chitosan and CACs may be a good strategy for promoting a pro-angiogenic ectopic site for islet transplantation. Yet, the results also highlight the need to select appropriate models in order to effectively evaluate vascularization strategies in the context of diabetes.

## Supporting Information

File S1 **This file contains the Supplemental materials and methods, Figure S1, Figure S2, Figure S3, Figure S4, Figure S5, Table S1, Table S2, Table S3, Table S4, Table S5, Table S6, Table S7, and the Supplemental references.**
(PDF)
