# Supplementary material for: Evaluation of a Collagen-Chitosan Hydrogel for Potential Use as a Pro-Angiogenic Site for Islet Transplantation
Source: PLoS One. 2013 Oct 18;8(10):e77538. doi: 10.1371/journal.pone.0077538 (PMC3799615; doi:10.1371/journal.pone.0077538)
Supplement: File S1 — This file contains the Supplemental materials and methods, Figure S1, Figure S2, Figure S3, Figure S4, Figure S5, Table S1, Table S2, Table S3, Table S4, Table S5, Table S6, Table S7, and the Supplemental references. (PDF) [file pone.0077538.s001.pdf]

SUPPLEMENTAL File S1

McBane et al. (2013) PLoS One

Supplemental Materials and Methods

Critical Point Drying (CPD) and Scanning Electron Microscopy (SEM)

Collagen and collagen-chitosan hydrogels (8mm disks) were fixed in 3% glutaraldehyde and dehydrated by incubating them in increasing concentrations of ethanol prior to CPD (Emitech K850) using CO_2 as the transitional fluid at a temperature of 31.1°C and a pressure of 1072 psi. Prior to SEM, samples were fractured using liquid nitrogen and sputter coated (Anatech, Hummer VII) with an ultra thin coating (3nm) of a 40:60 gold/palladium alloy to avoid charging effects. Samples were imaged by SEM (VegaII XMU; Tescan, Czech Republic) using two types of detectors; a back scattering electron detector (BSE) and a secondary detector (SE) and processed using Vega TC software (Tescan). To avoid damaging the samples, a low accelerating voltage of 8kV was applied. Fiber length, fiber diameter and number of fiber intersections (cross-links) were quantified by evaluating representative images from 4 separate samples using Image-J software.

Mechanical Testing

Hydrogels were swelled for 48 hours in PBS at 37°C. Samples (8mm disks) were measured for thickness (>3mm for tested sample) and placed in PBS for an additional 1h prior to mechanical testing. Unconfined compression tests were performed using a servo-hydraulic material testing system (MTS Bionix 858) equipped with a 5kg load cell. Crosshead position and load were recorded using Instron Wavemaker software at a cross speed of 50%/min and strained to a maximum of 65% strain. Ten specimens were tested for each type of hydrogel. For mechanical strain, the data obtained was converted to engineering stress-engineering strain, using sample thickness, cross-sectional area and initial gauge length. The stress-strain data was fitted for each individual sample to a five-parameter double exponential growth model using:

$$\sigma = y_0 + a \cdot exp(b \cdot \varepsilon) + c \cdot exp(d \cdot \varepsilon) \qquad \text{Equation (1)}$$

where σ is stress, ε is strain, and y_0, a, b, c and d are curve fitting parameters. The elastic modulus, as a function of strain was calculated by differentiating Equation (1) as follows:

$$\sigma' = a \cdot b \cdot exp(b \cdot \varepsilon) + c \cdot d \cdot exp(d \cdot \varepsilon) \qquad \text{Equation (2)}$$

where σ' is the tangent modulus, ε is strain, and a, b, c and d are curve fitting parameters. The elastic modulus was calculated in the linear region of the stress-strain curve.

Histology

Hydrogels were explanted and fixed in 4% paraformaldehyde overnight at 4°C and dehydrated using 15% and 30% sucrose (in PBS). The samples were then embedded in OCT (Sakura Finetek) and snap frozen with liquid nitrogen. Samples were kept at -80°C and then sections (10µm) were prepared for hematoxylin phloxine saffron (HPS) staining or immunofluorescence (described below).

Immunofluorescence

Sections were blocked with 5% bovine serum albumin (for CXCR4) or 50% normal horse serum (for von Willebrand factor (vWF)) and incubated with primary antibodies against CXCR4 (1:50; Abcam) for angiogenic cells or vWF (1:50, Abcam) for endothelial cells. Slides were then washed and incubated with appropriate secondary antibodies (1:600) conjugated to Texas Red. Slides were then mounted with 4′,6-diamido-2-phenylindole (DAPI) mounting medium (Vector Labs Inc.) and imaged using a Zeiss fluorescence microscope.

Table S1. Summary of key cytokines involved in angiogenesis and islet graft survival.

Cytokine	Classification	Effect on angiogenesis	Reference	Effects on islet/graft survival	Reference
**BLC**	Chemokine	-	[1]	-	[2]
**GM-CSF**	Growth factor	+	[3]	+	[4]
**IFN-γ**	Pro-inflammatory cytokine	+/-	[5]	-	[4,6,7,8,9,10]
**IL-1β**	Pro-inflammatory cytokine	+	[11]	-	[6,10]
**IL-12p70**	Pro-inflammatory cytokine	-	[12,13]	-	[8]
**Lymphotactin**	Chemokine	-	[14]	-	[15]
**MCP-1, MCP-5**	Chemokines	+	[16,17,18]	-	[15,19,20,21]
**MIG**	Chemokine	-	[22,23]	-	[6]
**PF-4**	Chemokine	-	[1,23,24,25]	-	[25]
**RANTES**	Chemokine	+	[26]	-	[15,27,28,29]
**SCF**	Growth factor	+	[30,31]	+	[32]
**SDF-1α**	Chemokine	+	[33,34,35,36]	+	[37]
**TARC**	Chemokine	+	[38]	-	[39]
**TNF-α**	Pro-inflammatory cytokine	+ (low dose)	[40,41,42]	-	[7,10]
**VCAM-1**	Adhesion molecule	+	[41,43]	+	[44]
**VEGF**	Growth factor	+	[40,45,46,47]	+	[7,44,48,49,50,51]

BLC: B-lymphocyte chemoattractant; GM-CSF: granulocyte macrophage-colony stimulating factor; IFN-γ: interferon-γ; IL: interleukin; MCP: monocyte chemotactic protein; MIG: monokine induced by interferon-γ; MIP: monocyte inflammatory protein; PF-4: platelet factor-4; RANTES: regulated upon activation, normal T-cell expressed and secreted; SCF: stem cell factor; SDF-1α: stromal cell derived factor-1α; TARC: thymus and activation regulated chemokine; TNF-α: tumour necrosis factor-α; VCAM-1: vascular cell adhesion molecule-1; VEGF: vascular endothelial growth factor.

Supplemental Results

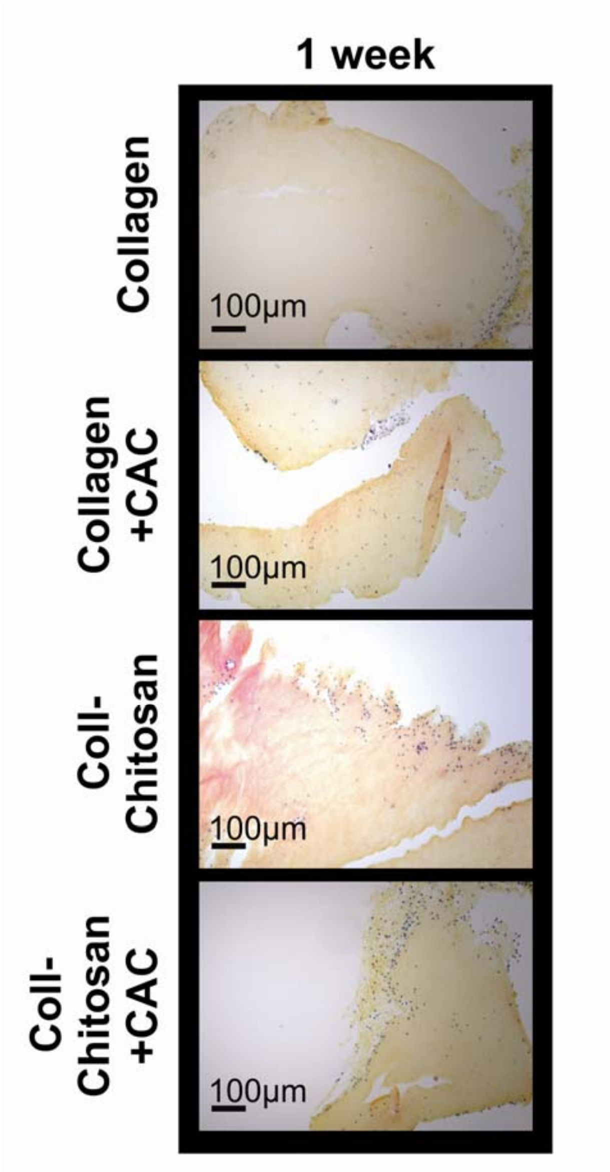

Figure S1. Representative images of hematoxylin phloxine saffron-stained implants at 1 week.

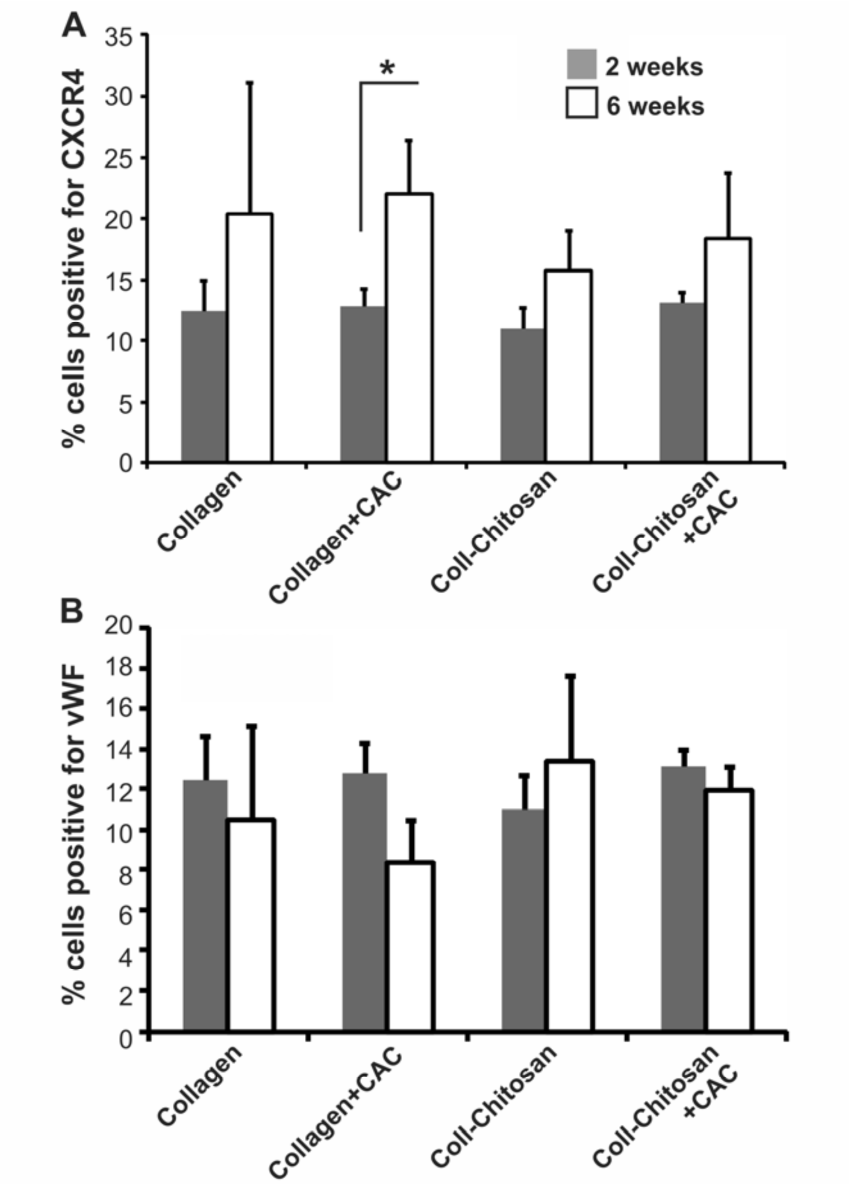

Figure S2. Quantification of $CXCR4^+$ and vWF^+ cells in subcutaneous implants from diabetic mice. Quantification of $CXCR4^+$ (A) and vWF^+ (B) cells in subcutaneously implanted hydrogels explanted after 2 or 6 weeks (***p<0.05).

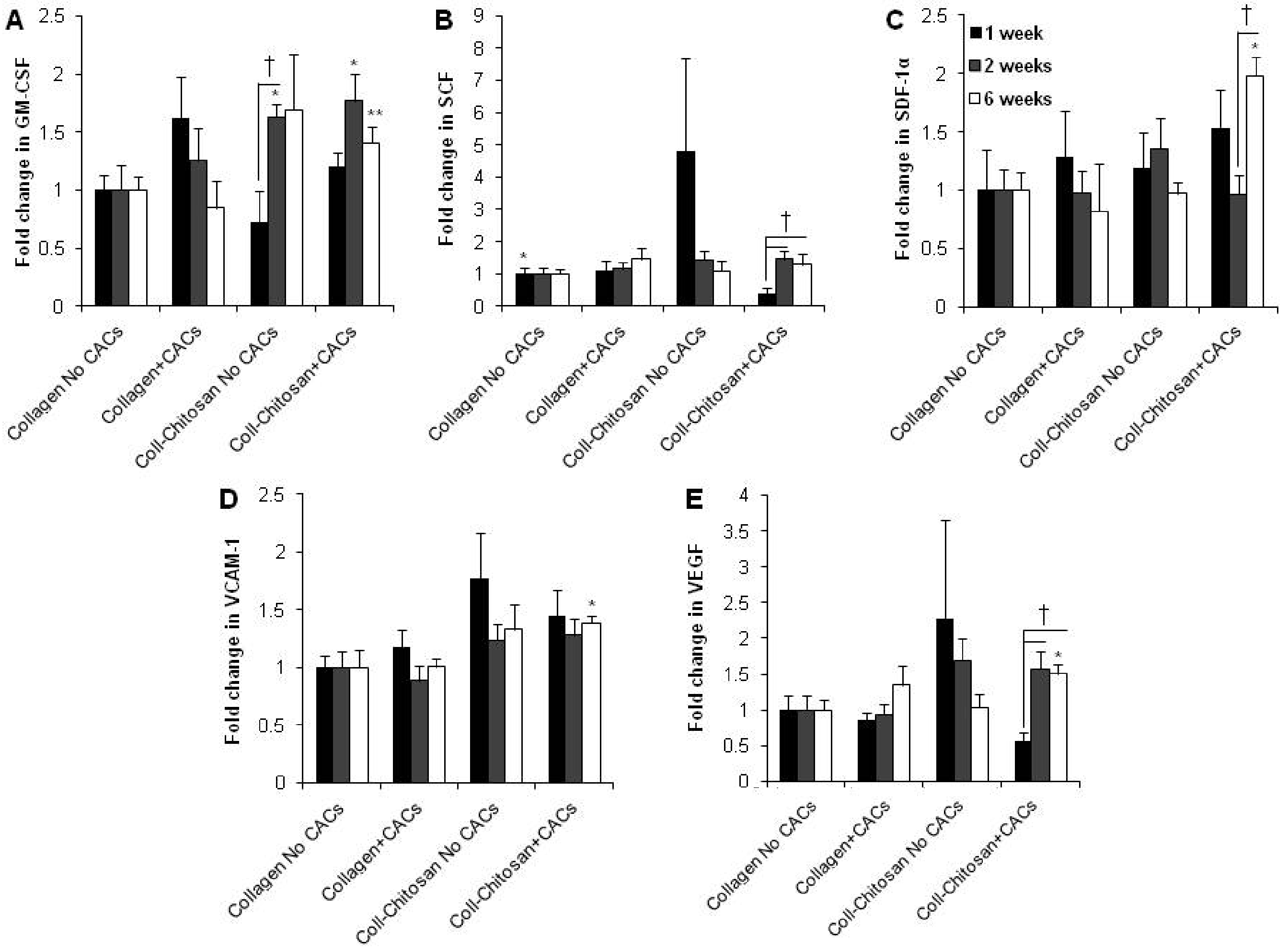

Figure S3. Expression of pro-angiogenic/pro-islet cytokines in subcutaneous implants from non-diabetic mice. The expression of GM-CSF **(A)**, SCF **(B)**, SCF-1α **(C)**, VCAM-1 **(D)**, and VEGF **(E)** protein in hydrogels implanted for 1 (black bars), 2 (gray bars) and 6 weeks (white bars) was normalized to the levels in the collagen hydrogel at their respective time point (n=3 for 1 and 6 weeks; n=5 for 2 weeks). P-values in **(A)**: *p=0.017 and p=0.018 for collagen-chitosan and collagen-chitosan+CACs vs. collagen at 2 weeks, respectively; **p=0.032 vs. collagen at 6 weeks; †p=0.012. In **(B)**: *p=0.023 vs. collagen-chitosan+CAC at 1 week; †p=0.006 and p=0.04 for 2 week and 6 week vs. 1 week in collagen-chitosan+CAC implants. In **(C)**: *p=0.0008, p=0.023 and p=0.0004 vs. collagen, collagen+CAC, and collagen-chitosan, respectively at 6 weeks; †p=0.0007. In **(D)**: *p=0.043 vs. collagen at 6 weeks. In **(E)**: *p=0.015 vs. collagen at 6 weeks; †p=0.006 and p=0.00013 for 2 and 6 weeks vs. 1 week, respectively.

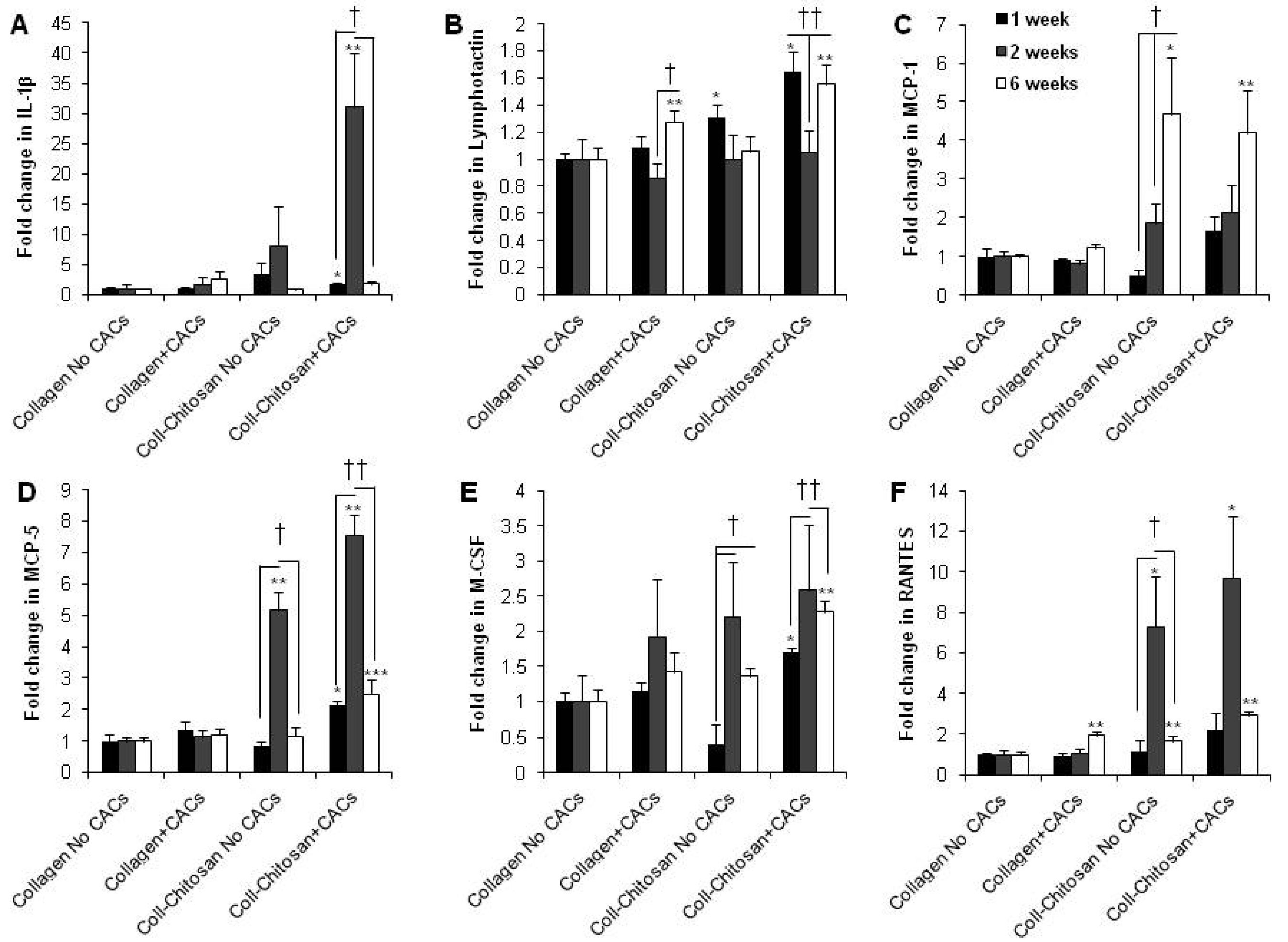

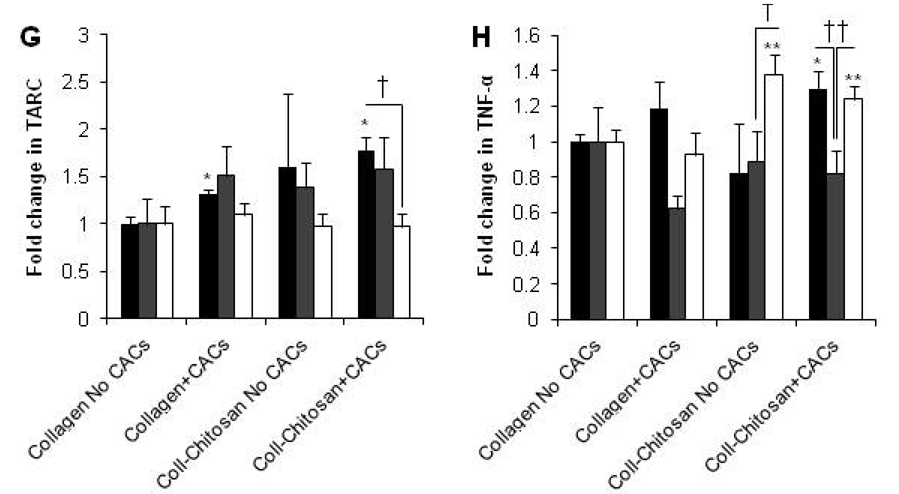

Figure S4. Expression of pro-angiogenic/anti-islet cytokines in implants from non-diabetic mice. The expression of IL-1β **(A)**, lymphotactin **(B)**, MCP-1 **(C)**, MCP-5 **(D)**, M-CSF **(E)**, RANTES **(F)**, TARC **(G)** and TNF-α **(H)** protein in hydrogels implanted for 1, 2 and 6 weeks was normalized to collagen hydrogel levels at their respective time point (n=3 for 1 and 6 weeks; n=5 for 2 weeks). P-values in **(A)**: *p=0.016 vs. collagen at 1 week; **$p\leq$0.044 vs. all others at 2 weeks; $^{\dagger}p$=0.007. In **(B)**: *$p\leq$0.014 vs. collagen at 1 week; **$p\leq$0.04 vs. collagen at 6 weeks; $^{\dagger}p$=0.007; $^{\dagger\dagger}p\leq$0.025. In **(C)**: *p=0.043 vs. collagen at 1 week; **$p\leq$0.03 vs. collagen and collagen+CAC at 6 weeks; $^{\dagger}p\leq$0.03. In **(D)**: *$p\leq$0.036 vs. all other implants at 1 week; **p<0.0001 vs. collagen and collagen+CAC at 2 weeks; ***$p\leq$0.027 vs. all other implants at 6 weeks; $^{\dagger}p$<0.0001; $^{\dagger\dagger}p\leq$0.0007. In **(E)**: *$p\leq$0.0030 vs. all other implants at 1 week; **$p\leq$0.021 vs. all other implants at 6 weeks; $^{\dagger}p\leq$0.011; $^{\dagger\dagger}p$=0.008. In **(F)**: *$p\leq$0.027 vs. collagen and collagen+CAC at 2 weeks; **$p\leq$0.047 vs. collagen at 6 weeks; $^{\dagger}p\leq$0.041. In **(G)**: *$p\leq$0.009 vs. collagen at 1 week; $^{\dagger}p$=0.0008. In **(H)**: *p=0.026 vs. collagen at 1 week; **$p\leq$0.049 vs. collagen and collagen+CAC implants at 6 weeks; $^{\dagger}p$=0.025; $^{\dagger\dagger}p\leq$0.016.

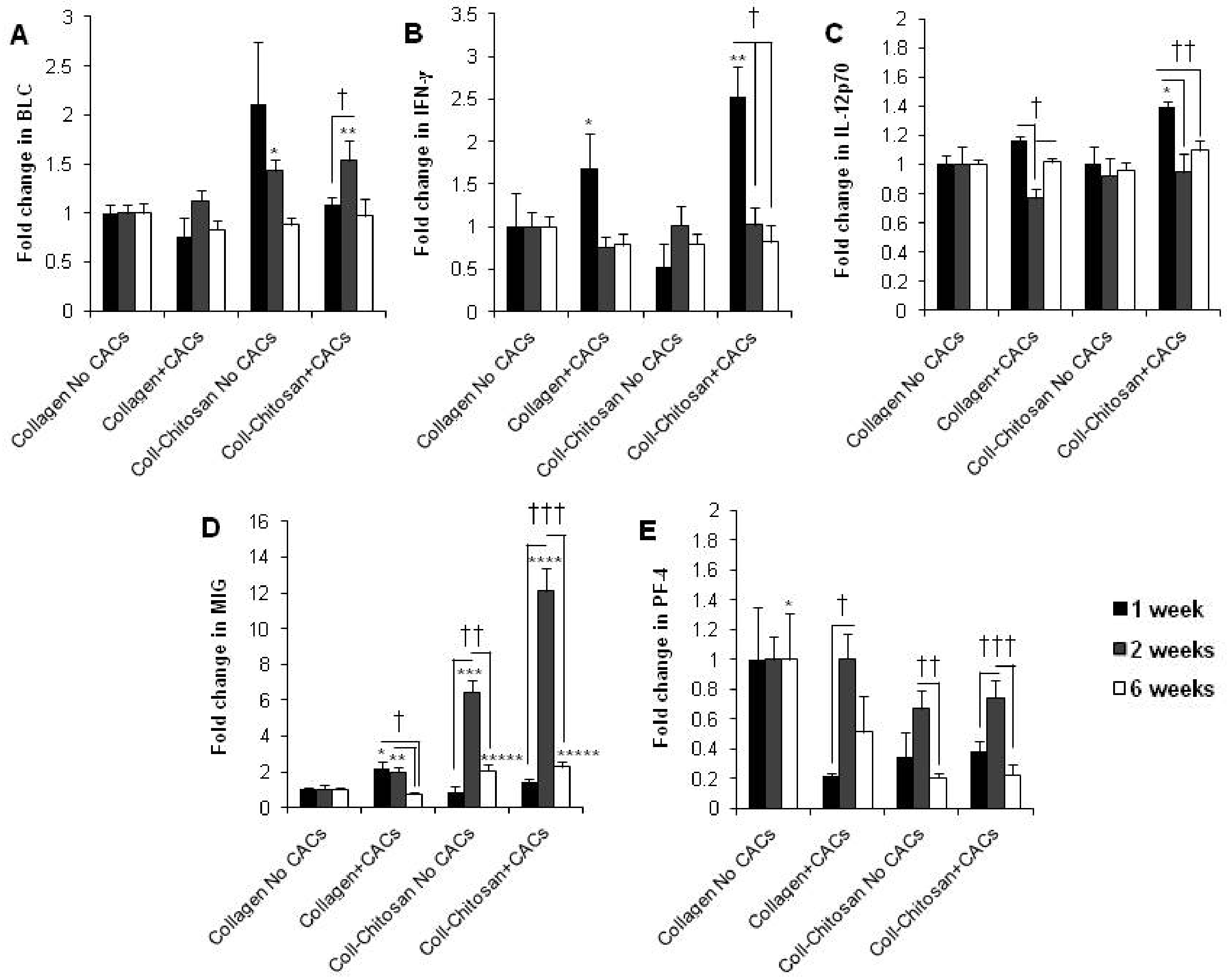

Figure S5. Expression of anti-angiogenic/anti-islet cytokines in subcutaneous implants from non-diabetic mice. The expression of BLC **(A)**, IFN-λ **(B)**, IL12p70 **(C)**, MIG **(D)**, and PF-4 **(E)** protein in hydrogels implanted for 1 (black bars), 2 (gray bars) and 6 weeks (white bars) was normalized to the levels in the collagen hydrogel at their respective time point (n=3 for 1 and 6 weeks; n=5 for 2 weeks). P-values in **(A)**: *$p \leq 0.031$ vs. collagen and collagen+CAC implants at 2 weeks; **$p=0.023$ vs. collagen at 2 weeks; $^{\dagger}p=0.049$. In **(B)**: *$p=0.033$ vs. collagen-chitosan at 1 week; **$p \leq 0.012$ vs. collagen and collagen-chitosan implants at 1 week; $^{\dagger}p=0.0039$. In **(C)**: *$p=0.043$ vs. collagen at 1 week; $^{\dagger}p \leq 0.0053$; $^{\dagger\dagger}p \leq 0.0048$. In **(D)**: *$p \leq 0.039$ vs. collagen and collagen-chitosan at 1 week; **$p=0.016$ vs. collagen at 2 weeks; ***$p \leq 0.00017$ vs. collagen and collagen+CAC at 2 weeks; ****$p \leq 0.0013$ vs. all other implants at 2 weeks; *****$p \leq 0.027$ vs. collagen and collagen+CAC implants at 6 weeks; $^{\dagger}p \leq 0.019$; $^{\dagger\dagger}p<0.0001$; $^{\dagger\dagger\dagger}p \leq 0.00087$. In **(E)**: *$p \leq 0.038$ collagen-chitosan and collagen-chitosan+CAC at 6 weeks; $^{\dagger}p=0.0003$; $^{\dagger\dagger}p=0.0027$; $^{\dagger\dagger\dagger}p \leq 0.025$.

Table S2. Relative cytokine array values for pro-angiogenic/pro-islet proteins from collagen hydrogel implants in diabetic mice at 1, 2, and 6 weeks.

	Average intensity/mg protein at 1 week (±SD)	Average intensity/mg protein at 2 week (±SD)	Average % remaining at 2 weeks vs. 1 week	Average intensity/mg protein at 6 week (±SD)	Average % remaining at 6 weeks vs. 1 week
**GM-CSF**	0.06±0.05	0.23±0.31	410.4±591.0	0.22±0.06	375.3±100.3
**SCF**	0.36±0.34	0.050±0.057	14.2±17.8	0.078±0.055	22.0±12.0
**SDF-1α**	0.37±0.48	0.046±0.059	12.3±17.4	0.13±0.03	34.3±7.7
**VCAM-1**	11.1±3.9	11.1±6.4	100.4±17.4	19.9±1.2	180.0±11.0
**VEGF**	0.34±0.48	0.15±0.18	44.7±58.7	0.096±0.057	28.3±16.8

SD=Standard deviation. n=3 except for 6 week samples which are n=2.

Table S3. Relative cytokine array values for pro-angiogenic/anti-islet proteins from collagen hydrogel implants in diabetic mice at 1, 2, and 6 weeks.

	Average intensity/mg protein at 1 week±SD	Average intensity/mg protein at 2 week±SD	Average % remaining at 2 weeks vs. 1 week±SD	Average intensity/mg protein at 6 week±SD	Average % remaining at 6 weeks vs. 1 week±SD
**IL-1β**	0.57±0.75	0.10±0.04	18.3±8.2	0.1±0.06	17.3±10.8
**Lymphotactin**	0.28±0.08	0.28±0.07	86.1±122.3	0.37±0.09	130.1±31.1
**MCP-1**	0.51±0.16	0.51±0.15	67.0±30.4	0.88±0.18	171.9±35.1
**MCP-5**	1.06±0.17	0.72±1.00	67.8±105.8	0.61±0.20	57.6±19.1
**M-CSF**	0.13±0.03	0.11±0.07	84.6±54.7	0.33±0.08	259.2±64.3
**RANTES**	0.56±0.72	0.34±0.46	60.5±93.4	0.19±0.03	34.6±6.1
**TARC**	0.27±0.23	0.18±0.22	66.4±92.1	0.29±0.18	106.6±65.5
**TNF-α**	0.58±0.05	0.38±0.28	65.0±52.9	0.47±0.03	80.1±4.6

SD=Standard deviation. n=3 except for 6 week samples where n=2.

Table S4. Relative cytokine array values for anti-angiogenic/anti-islet proteins from collagen hydrogel implants in diabetic mice at 1, 2, and 6 weeks.

	Average intensity/mg protein at 1 week (±SD)	Average intensity/mg protein at 2 week±SD	Average % remaining at 2 weeks vs. 1 week±SD	Average intensity/mg protein at 6 week±SD	Average % remaining at 6 weeks vs. 1 week±SD
**BLC**	11.3±10.3	6.77±9.49	60.1±94.0	1.71±0.31	15.1±2.7
**IFN-γ**	0.07±0.04	0.39±0.50	568.6±812.6	0.14±0.04	210.4±63.6
**IL-12 p70**	0.61±0.12	0.86±0.65	140.4±118.1	1.34±0.08	219.4±13.2
**MIG**	0.13±0.06	0.28±0.29	214.5±246.6	0.37±0.05	282.5±36.8
**PF-4**	1.83±1.97	4.63±5.56	252.6±339.4	1.04±0.58	57.0±31.7

SD=Standard deviation. n=3 except for 6 week samples where n=2.

Table S5. Relative cytokine array values for pro-angiogenic/pro-islet proteins from collagen hydrogel implants in non-diabetic mice at 1, 2, and 6 weeks.

	Average intensity/mg protein at 1 week (±SE)	Average intensity/mg protein at 2 weeks* (±SE)	Average % remaining at 2 weeks* vs. 1 week (±SE)	Average intensity/mg protein at 6 week (±SE)	Average % remaining at 6 weeks vs. 1 week (±SE)
**GM-CSF**	0.079±0.023	0.13±0.08	157.1±33.5	0.16±0.04	203.4±22.6
**SCF**	0.061±0.025	0.094±0.049	153.4±26.6	0.081±0.027	132.9±19.6
**SDF-1α**	0.052±0.040	0.16±0.09	306.9±54.5	0.076±0.027	146.2±23.0
**VCAM-1**	11.38±2.58	24.8±3.6	218.2±31.7	18.4±6.4	161.4±25.0
**VEGF**	0.17±0.08	0.17±0.11	99.5±20.8	0.12±0.02	66.4±9.46

SE=Standard error. n=3 except for 2 week samples which are *n=5.

Table S6. Relative cytokine array values for pro-angiogenic/anti-islet proteins from collagen hydrogel implants in non-diabetic mice at 1, 2, and 6 weeks at 1, 2, and 6 weeks.

	Average intensity/mg protein at 1 week (±SE)	Average intensity/mg protein at 2 weeks* (±SE)	Average % remaining at 2 weeks* vs. 1 week (±SE)	Average intensity/mg protein at 6 week (±SE)	Average % remaining at 6 weeks vs. 1 week (±SE)
**IL-1β**	0.18±0.03	0.003±0.002	1.79±1.27	0.30±0.13	169.2±78.0
**Lymphotactin**	0.26±0.01	0.55±0.08	209.5±30.5	0.39±0.04	150.9±13.4
**MCP-1**	1.16±0.27	0.55±0.07	47.0±6.1	0.84±0.04	72.1±3.79
**MCP-5**	1.22±0.28	0.30±0.04	24.9±3.1	0.60±0.06	49.0±4.9
**M-CSF**	0.23±0.03	0.081±0.031	34.6±13.1	0.30±0.05	128.6±21.8
**RANTES**	0.22±0.03	0.19±0.05	89.6±20.7	0.13±0.02	61.3±10.5
**TARC**	0.15±0.01	0.11±0.03	71.5±19.3	0.26±0.05	170.5±30.8
**TNF-α**	0.55±0.02	0.99±0.19	178.6±34.7	0.43±0.03	78.2±5.6

SE=Standard error. n=3 except for 2 week samples which are *n=5.

Table S7. Relative cytokine array values for anti-angiogenic/anti-islet proteins from collagen hydrogel implants in non-diabetic mice at 1, 2, and 6 weeks.

	Average intensity/mg protein at 1 week (±SE)	Average intensity/mg protein at 2 weeks* (±SE)	Average % remaining at 2 weeks* vs. 1 week (±SE)	Average intensity/mg protein at 6 weeks (±SE)	Average % remaining at 6 weeks vs. 1 week (±SE)
**BLC**	6.47±0.57	0.23±0.02	3.54±0.29	2.07±0.20	32.0±3.0
**IFN-γ**	0.058±0.023	0.64±0.11	1102.5±193.5	0.26±0.03	445.5±55.7
**IL-12 p70**	0.64±0.04	0.83±0.10	129.8±16.2	1.29±0.05	201.5±7.3
**MIG**	0.12±0.02	0.072±0.019	60.9±16.1	0.35±0.02	294.1±20.4
**PF-4**	5.42±1.87	3.36±0.51	61.9±9.4	5.00±1.53	92.2±28.1

SE=Standard error. *n=3 except for 2 week samples which are n=5.

Supplemental References

1. Spinetti G, Camarda G, Bernardini G, Romano Di Peppe S, Capogrossi MC, et al. (2001) The chemokine CXCL13 (BCA-1) inhibits FGF-2 effects on endothelial cells. Biochem Biophys Res Commun 289: 19-24.
2. Mao Y, Wang M, Zhou Q, Jin J, Wang Y, et al. (2011) CXCL10 and CXCL13 Expression were highly up-regulated in peripheral blood mononuclear cells in acute rejection and poor response to anti-rejection therapy. J Clin Immunol 31: 414-418.
3. Wang QR, Wang F, Zhu WB, Lei J, Huang YH, et al. (2009) GM-CSF accelerates proliferation of endothelial progenitor cells from murine bone marrow mononuclear cells in vitro. Cytokine 45: 174-178.
4. Enzler T, Gillessen S, Dougan M, Allison JP, Neuberg D, et al. (2007) Functional deficiencies of granulocyte-macrophage colony stimulating factor and interleukin-3 contribute to insulitis and destruction of beta cells. Blood 110: 954-961.
5. Zaidi MR, Merlino G (2011) The two faces of interferon-gamma in cancer. Clin Cancer Res 17: 6118-6124.
6. Cardozo AK, Proost P, Gysemans C, Chen MC, Mathieu C, et al. (2003) IL-1beta and IFN-gamma induce the expression of diverse chemokines and IL-15 in human and rat pancreatic islet cells, and in islets from pre-diabetic NOD mice. Diabetologia 46: 255-266.
7. Park KS, Kim YS, Kim JH, Choi BK, Kim SH, et al. (2009) Influence of human allogenic bone marrow and cord blood-derived mesenchymal stem cell secreting trophic factors on ATP (adenosine-5'-triphosphate)/ADP (adenosine-5'-diphosphate) ratio and insulin secretory function of isolated human islets from cadaveric donor. Transplant Proc 41: 3813-3818.
8. Matsuoka N, Itoh T, Watarai H, Sekine-Kondo E, Nagata N, et al. (2010) High-mobility group box 1 is involved in the initial events of early loss of transplanted islets in mice. J Clin Invest 120: 735-743.
9. Yamada K, Otabe S, Inada C, Takane N, Nonaka K (1993) Nitric oxide and nitric oxide synthase mRNA induction in mouse islet cells by interferon-gamma plus tumor necrosis factor-alpha. Biochem Biophys Res Commun 197: 22-27.
10. Yang Z, Chen M, Ellett JD, Carter JD, Brayman KL, et al. (2005) Inflammatory blockade improves human pancreatic islet function and viability. Am J Transplant 5: 475-483.
11. Qin SL, Li TS, Takahashi M, Hamano K (2006) In vitro assessment of the effect of interleukin-1beta on angiogenic potential of bone marrow cells. Circ J 70: 1195-1199.
12. Piazzolla G, Tortorella C, Fiore G, Fanelli M, Pisconti A, et al. (2001) Interleukin-12 p40/p70 ratio and in vivo responsiveness to IFN-alpha treatment in chronic hepatitis C. J Interferon Cytokine Res 21: 453-461.
13. Yasuda H, Nagata M, Arisawa K, Yoshida R, Fujihira K, et al. (1998) Local expression of immunoregulatory IL-12p40 gene prolonged syngeneic islet graft survival in diabetic NOD mice. J Clin Invest 102: 1807-1814.
14. Zhang J, Zhou Z, Wang C, Shen J, Zheng Y, et al. (2011) Reduced tumorigenesis of EG7 after interleukin-10 gene transfer and enhanced efficacy in combination with intratumorally injection of adenovirus-mediated lymphotactin and the underlying mechanism. Cancer Immunol Immunother 60: 559-573.
15. Bradley LM, Asensio VC, Schioetz LK, Harbertson J, Krahl T, et al. (1999) Islet-specific Th1, but not Th2, cells secrete multiple chemokines and promote rapid induction of autoimmune diabetes. J Immunol 162: 2511-2520.
16. Tsui P, Das A, Whitaker B, Tornetta M, Stowell N, et al. (2007) Generation, characterization and biological activity of CCL2 (MCP-1/JE) and CCL12 (MCP-5) specific antibodies. Hum Antibodies 16: 117-125.

17. Nesbit M, Schaider H, Miller TH, Herlyn M (2001) Low-level monocyte chemoattractant protein-1 stimulation of monocytes leads to tumor formation in nontumorigenic melanoma cells. J Immunol 166: 6483-6490.
18. Zhang Y, Ingram DA, Murphy MP, Saadatzadeh MR, Mead LE, et al. (2009) Release of proinflammatory mediators and expression of proinflammatory adhesion molecules by endothelial progenitor cells. Am J Physiol Heart Circ Physiol 296: H1675-1682.
19. Melzi R, Mercalli A, Sordi V, Cantarelli E, Nano R, et al. (2010) Role of CCL2/MCP-1 in islet transplantation. Cell Transplant 19: 1031-1046.
20. Piemonti L, Leone BE, Nano R, Saccani A, Monti P, et al. (2002) Human pancreatic islets produce and secrete MCP-1/CCL2: relevance in human islet transplantation. Diabetes 51: 55-65.
21. Bertuzzi F, Marzorati S, Maffi P, Piemonti L, Melzi R, et al. (2004) Tissue factor and CCL2/monocyte chemoattractant protein-1 released by human islets affect islet engraftment in type 1 diabetic recipients. J Clin Endocrinol Metab 89: 5724-5728.
22. Moore BB, Arenberg DA, Strieter RM (1998) The role of CXC chemokines in the regulation of angiogenesis in association with lung cancer. Trends Cardiovasc Med 8: 51-58.
23. Strieter RM, Polverini PJ, Arenberg DA, Kunkel SL (1995) The role of CXC chemokines as regulators of angiogenesis. Shock 4: 155-160.
24. Shao XJ, Xie FM (2005) Influence of angiogenesis inhibitors, endostatin and PF-4, on lymphangiogenesis. Lymphology 38: 1-8.
25. Vandercappellen J, Van Damme J, Struyf S (2011) The role of the CXC chemokines platelet factor-4 (CXCL4/PF-4) and its variant (CXCL4L1/PF-4var) in inflammation, angiogenesis and cancer. Cytokine Growth Factor Rev 22: 1-18.
26. Suffee N, Richard B, Hlawaty H, Oudar O, Charnaux N, et al. (2011) Angiogenic properties of the chemokine RANTES/CCL5. Biochem Soc Trans 39: 1649-1653.
27. Solomon MF, Kuziel WA, Mann DA, Simeonovic CJ (2003) The role of chemokines and their receptors in the rejection of pig islet tissue xenografts. Xenotransplantation 10: 164-177.
28. Solomon MF, Kuziel WA, Simeonovic CJ (2004) The contribution of chemokines and chemokine receptors to the rejection of fetal proislet allografts. Cell Transplant 13: 503-514.
29. Itoh T, Sugimoto K, Takita M, Shimoda M, Chujo D, et al. (2012) Low temperature condition prevents hypoxia induced islet cell damage and HMGB1 release in a mouse model. Cell Transplant 21: 1367-1370.
30. Zhang W, Stoica G, Tasca SI, Kelly KA, Meininger CJ (2000) Modulation of tumor angiogenesis by stem cell factor. Cancer Res 60: 6757-6762.
31. Bouchentouf M, Forner K, Cuerquis J, Boulassel MR, Routy JP, et al. (2010) A novel and simplified method of culture of human blood derived early endothelial progenitor cells for the treatment of ischemic vascular disease. Cell Transplant 20: 1431-1443.
32. Wu Y, Li J, Saleem S, Yee SP, Hardikar AA, et al. (2010) c-Kit and stem cell factor regulate PANC-1 cell differentiation into insulin- and glucagon-producing cells. Lab Invest 90: 1373-1384.
33. Deshane J, Chen S, Caballero S, Grochot-Przeczek A, Was H, et al. (2007) Stromal cell-derived factor 1 promotes angiogenesis via a heme oxygenase 1-dependent mechanism. J Exp Med 204: 605-618.
34. Tang J, Wang J, Yang J, Kong X, Zheng F, et al. (2009) Mesenchymal stem cells over-expressing SDF-1 promote angiogenesis and improve heart function in experimental myocardial infarction in rats. Eur J Cardiothorac Surg 36: 644-650.
35. Hiasa K, Ishibashi M, Ohtani K, Inoue S, Zhao Q, et al. (2004) Gene transfer of stromal cell-derived factor-1alpha enhances ischemic vasculogenesis and angiogenesis via vascular endothelial growth factor/endothelial nitric oxide synthase-related pathway: next-generation chemokine therapy for therapeutic neovascularization. Circulation 109: 2454-2461.

36. Chu CY, Cha ST, Lin WC, Lu PH, Tan CT, et al. (2009) Stromal cell-derived factor-1alpha (SDF-1alpha/CXCL12)-enhanced angiogenesis of human basal cell carcinoma cells involves ERK1/2-NF-kappaB/interleukin-6 pathway. Carcinogenesis 30: 205-213.
37. Aboumrad E, Madec AM, Thivolet C (2007) The CXCR4/CXCL12 (SDF-1) signalling pathway protects non-obese diabetic mouse from autoimmune diabetes. Clin Exp Immunol 148: 432-439.
38. Stroo I, Stokman G, Teske GJ, Raven A, Butter LM, et al. (2010) Chemokine expression in renal ischemia/reperfusion injury is most profound during the reparative phase. Int Immunol 22: 433-442.
39. Huser N, Tertilt C, Gerauer K, Maier S, Traeger T, et al. (2005) CCR4-deficient mice show prolonged graft survival in a chronic cardiac transplant rejection model. Eur J Immunol 35: 128-138.
40. Jing Y, Ma N, Fan T, Wang C, Bu X, et al. (2011) Tumor necrosis factor-alpha promotes tumor growth by inducing vascular endothelial growth factor. Cancer Invest 29: 485-493.
41. Bouchentouf M, Forner KA, Cuerquis J, Michaud V, Zheng J, et al. (2010) Induction of cardiac angiogenesis requires killer cell lectin-like receptor 1 and alpha4beta7 integrin expression by NK cells. J Immunol 185: 7014-7025.
42. Sugano M, Tsuchida K, Makino N (2004) Intramuscular gene transfer of soluble tumor necrosis factor-alpha receptor 1 activates vascular endothelial growth factor receptor and accelerates angiogenesis in a rat model of hindlimb ischemia. Circulation 109: 797-802.
43. Buhring HJ, Treml S, Cerabona F, de Zwart P, Kanz L, et al. (2009) Phenotypic characterization of distinct human bone marrow-derived MSC subsets. Ann N Y Acad Sci 1176: 124-134.
44. Vivot K, Jeandidier N, Dollinger C, Bietiger W, Pinget M, et al. (2011) Role of islet culture on angiogenic and inflammatory mechanisms. Transplant Proc 43: 3201-3204.
45. Ferrara N (2004) Vascular endothelial growth factor: basic science and clinical progress. Endocr Rev 25: 581-611.
46. Ferrara N (1995) The role of vascular endothelial growth factor in pathological angiogenesis. Breast Cancer Res Treat 36: 127-137.
47. Leung DW, Cachianes G, Kuang WJ, Goeddel DV, Ferrara N (1989) Vascular endothelial growth factor is a secreted angiogenic mitogen. Science 246: 1306-1309.
48. Linn T, Erb D, Schneider D, Kidszun A, Elcin AE, et al. (2003) Polymers for induction of revascularization in the rat fascial flap: application of vascular endothelial growth factor and pancreatic islet cells. Cell Transplant 12: 769-778.
49. Chow LW, Wang LJ, Kaufman DB, Stupp SI (2010) Self-assembling nanostructures to deliver angiogenic factors to pancreatic islets. Biomaterials 31: 6154-6161.
50. Brissova M, Shostak A, Shiota M, Wiebe PO, Poffenberger G, et al. (2006) Pancreatic islet production of vascular endothelial growth factor--a is essential for islet vascularization, revascularization, and function. Diabetes 55: 2974-2985.
51. Sigrist S, Mechine-Neuville A, Mandes K, Calenda V, Legeay G, et al. (2003) Induction of angiogenesis in omentum with vascular endothelial growth factor: influence on the viability of encapsulated rat pancreatic islets during transplantation. J Vasc Res 40: 359-367.
